# Knowledge-Based Expert System for Orthopedic Injury Management With Integrated Transcutaneous Electrical Nerve Simulation Therapy

**DOI:** 10.1155/ijta/5878245

**Published:** 2025-06-23

**Authors:** Haneen Banjar, Shahad Almalki, Lama Almehmadi, Amjad Alshahrani, Ali Chaudhary, Reda Ghoname

**Affiliations:** ^1^Computer Science Department, Faculty of Computing and Information Technology, King Abdulaziz University, Jeddah, Saudi Arabia; ^2^Center of Research Excellence in Artificial Intelligence and Data Science, King Abdulaziz University, Jeddah, Saudi Arabia; ^3^Institute of Genomic Medicine Sciences, King Abdulaziz University, Jeddah, Saudi Arabia; ^4^Centre of Artificial Intelligence in Precision Medicines, King Abdulaziz University, Jeddah, Saudi Arabia; ^5^John Hopkins Aramco Healthcare, Dhahran, Saudi Arabia; ^6^Computer and Electrical Engineering Department, King Abdulaziz University, Jeddah, Saudi Arabia

**Keywords:** ankle injuries, ankle–foot orthoses (AFOs), knowledge-based expert system, personalized treatment, TENS therapy

## Abstract

Orthotic treatment, particularly for ankle injuries, often relies on devices such as ankle–foot orthoses (AFOs) to support and stabilize the foot and ankle. Given the high prevalence of lower extremity injuries, especially in sports, and the diagnostic challenges faced by clinicians, there is a pressing need for intelligent systems that aid in accurate assessment and personalized management. Misdiagnosis and suboptimal treatment of ankle conditions, including fractures and sprains, can lead to long-term complications and hinder recovery. This study presents *TeleOrtho*, a knowledge-based expert system designed to provide personalized diagnosis, treatment recommendations, and recovery plans for ankle injuries, integrating transcutaneous electrical nerve stimulation (TENS) therapy to enhance rehabilitation. The system was implemented using Flutter for cross-platform deployment and Python for expert rule modeling, and it features a user-friendly interface for both clinicians and patients. Diagnostic accuracy and treatment pathways were validated through two published case scenarios, confirming the system's alignment with medical guidelines. Static and dynamic testing demonstrated consistent functionality, and initial usability assessment indicated high user satisfaction (average SUS score: 85.3/100). The expert system reliably identified injury types based on structured symptom input and suggested individualized recovery plans. These results affirm the potential of TeleOrtho to reduce diagnostic errors, improve therapy adherence, and enhance clinical decision-making. Future work will focus on integrating imaging analysis, expanding the knowledge base to include additional orthopedic conditions, and conducting clinical trials for broader validation.

## 1. Introduction

Orthotics is a medical specialty that deals with the development and usage of orthoses. An orthosis is defined as “an externally applied device used to influence the structural and functional characteristics of the neuromuscular and skeletal system” [1]. The International Classification of Functioning, Disability, and Health (ICF) provides a comprehensive framework for describing human functioning in prosthetics and orthotics (P&O) clinical practice [2]. It can be used to demonstrate the impact of P&O devices on a person's functioning and to compile core sets of outcome measures for lower limb orthoses [2, 3]. The ICF covers various aspects of functioning, including body functions, structures, activities, and participation [2]. There are orthotics for the lower extremities, upper extremities, trunk, and head [1]. An orthosis that spans the ankle and foot is referred to as an ankle–foot orthosis (AFO). Lower extremity injuries are prevalent among athletes, accounting for over 50% of sports-related injuries [4]. Foot and ankle injuries, particularly sprains, strains, contusions, and fractures, are common in emergency departments and sports settings [5, 6]. A study of US high school athletes found that 52.8% of injuries were lower extremity-related, with football and soccer having the highest rates for boys and girls, respectively [6]. Ankle sprains were the most frequent injury, with an incidence of 206 per 100,000 [5]. The ankle, knee, and thigh were the most commonly injured areas, with girls experiencing a higher proportion of season-ending injuries compared to boys [6]. Misdiagnosis and undertreatment of foot and ankle injuries are common among healthcare providers, highlighting the need for improved awareness and management of these injuries [7]. According to Kolokotsios et al. [8], soccer participation has tripled over the last three decades, and as a result, more injuries have been reported during training or matches than in earlier decades. A variety of circumstances cause injuries. Long durations of time have been shown to cause weariness in athletes, leading to an increased risk of joint ankle injury. External and internal lateral ligaments (external and internal sprains), blood vessel injuries, high ankle sprains, and myotendinous injuries are the most prevalent injuries [8].

AFO injuries are evaluated during match and training times in a 10-year epidemiological study. The external lateral ligament was involved in 66.82% of all injuries between 2004 and 2009 (external ankle sprain). Meanwhile, internal lateral ligament (internal ankle sprain) injuries accounted for 8.71% of all injuries, blood vessel injuries accounted for 9.48%, and high ankle sprains accounted for 11.47% [8]. Thus, it is necessary to determine the causes and mechanisms of injuries in the foot area and treatment methods, including physical therapy, which rehabilitates the patient to lead his life normally again.

Physical therapy, also known as physiotherapy, is a healthcare profession focused on restoring and maximizing physical function, movement, and quality of life [9, 10]. It encompasses various interventions in areas such as promotion, prevention, treatment, and rehabilitation [11]. Physical therapists use evidence-based approaches to address a wide range of orthopedic and neurological conditions across different healthcare settings [10, 11]. Physical therapy also plays a crucial role in restoring movement, function, and overall health for individuals affected by injury, disease, or disability [12]. It involves physical examination, diagnosis, and intervention to promote health and prevent disease [12]. Physical therapists can address chronic diseases, which are the leading causes of morbidity and mortality, by promoting health-enhancing behaviors and lifestyle modifications [13]. Physical therapy is widely recommended by primary care providers for musculoskeletal conditions, offering various techniques to reduce pain and improve mobility [14, 15]. Treatments may include therapeutic exercises, manual techniques, and physical agents like heat or cold [14]. Additionally, it is possible to stimulate the body's natural painkillers with a transcutaneous electrical nerve stimulation (TENS) [16]. TENS is a nonpharmacological intervention used for pain management. It works by activating descending inhibitory systems in the central nervous system to reduce hyperalgesia [17]. TENS has shown effectiveness in reducing postoperative pain during movement and improving walking function when used as a supplement to pharmacological analgesia [18]. The mechanism of TENS involves the release of endogenous opioids and is supported by the gate control theory [19].

Despite the benefits of traditional orthopedic casts and physical therapy, several gaps exist in current treatments. Patients often experience prolonged recovery times, muscle atrophy, and decreased mobility due to the immobilization of the injured area. Additionally, the time-consuming nature of physical therapy sessions and the lack of personalized treatment plans can lead to suboptimal outcomes and patient noncompliance. Physical therapy compliance is a significant challenge for patients, with time constraints and frequent visits being major barriers [20]. Only 29% of patients fully adhere to at-home exercises, while 54% partially comply, performing only 33% of recommended repetitions [21]. Factors such as agitation, cognitive impairment, fatigue, and pain can impede rehabilitation success and participation during post-traumatic amnesia [22]. To address these issues, therapists often adopt an eclectic approach, combining interventions to remediate impairments and compensate for functional limitations [23]. Inpatient rehabilitation typically involves daily therapy sessions lasting about 38 min, focusing on gait and prefunctional activities [23]. To improve adherence, researchers suggest tailoring session duration to patient needs, conducting therapy earlier in the day, and developing specific solutions to increase motivation [21, 22].

This study is aimed at using artificial intelligence to develop an application and prototype that assists orthotics and patients in diagnosing and treating orthopedic injuries, particularly AFO injuries. To achieve this aim, the study objectives are divided into two main parts.

First, the study is aimed at building a knowledge-based expert system that includes several components: initial diagnosis results, treatment recommendations, recovery plans, and follow-up of the patient's condition during the treatment period. This expert system will utilize a comprehensive knowledge base of medical information related to the diagnosis and treatment of AFO injuries.

Second, the study will focus on building a simulation and prototype for the device. This includes creating a 3D model to represent the prototype's simulation and integrating a personalized TENS machine with the orthopedic cast to support the healing process. The prototype will be designed to maintain muscle activity and facilitate recovery.

To achieve these goals, the specific objectives of the study are as follows: identify and collect medical knowledge related to the diagnosis and treatment of AFO injuries to create a knowledge base, develop an expert system for diagnosing patients and making treatment recommendations, implement a mobile application that automates the process of injury diagnosis and follow-up of the patient's condition during the treatment period, and build a 3D model to simulate the prototype for the treatment of AFO injuries and maintain muscle activity.

The significance of integrating a knowledge-based expert system with personalized TENS treatment lies in its potential to revolutionize orthopedic care. By providing customized pain management solutions and real-time monitoring, this approach can significantly enhance patient outcomes. Patients will benefit from reduced pain, quicker recovery times, and improved mobility. Additionally, healthcare providers will be able to deliver more efficient and effective care, leading to better patient satisfaction and overall health outcomes. This innovative approach is aimed at streamlining the diagnostic and treatment process, reducing the burden on both patients and healthcare systems, and paving the way for advanced, AI-driven healthcare solutions.

### 1.1. Medical Overview

Ankle orthotics are essential devices used to support and stabilize the ankle joint, especially after injuries like sprains and fractures. They can be either prefabricated or custom-made, tailored to the patient's needs. These devices are vital in various situations, including preventing reinjury, aiding rehabilitation, and managing chronic instability. They are particularly crucial in early intervention to prevent further damage and promote healing. The choice of an orthotic device depends on the specific clinical indication and the patient's condition [24]. There are multiple types of ankle orthotics, each serving a unique purpose. For example, ankle air stirrups are useful for managing fractures, while elastic ankle sleeves are beneficial during the acute and rehabilitative stages of injuries. Other devices, such as lace-up braces and orthopedic cast braces, offer support for both acute injuries and chronic conditions. The classification of these devices as durable medical equipment (DME) emphasizes their importance in treatment protocols [24]. Additionally, these orthotics can significantly improve the quality of life for patients with chronic conditions by providing necessary support and stability. AFOs and knee–ankle–foot orthoses (KAFOs) provide comprehensive support extending above the ankle, making them suitable for severe conditions that require significant immobilization. These devices differ from simple foot orthotics, as they offer more extensive support, including to the knee and thigh, in the case of KAFOs. This added support is crucial for managing more severe injuries or congenital deformities [24].

Immobilization through casts or splints is a common treatment for various injuries, including fractures and severe soft-tissue injuries. These methods stabilize the affected area, aiding in proper healing. However, they come with potential complications such as compartment syndrome, ischemia, and skin issues. Careful monitoring and patient education are essential to prevent these complications. Proper application and timely intervention are essential to minimize these risks [25, 26].

Splinting offers several advantages over casting, including easier application and adjustability. Splints allow for natural swelling and reduce the risk of pressure-related complications, making them ideal for acute care settings. They are also beneficial in situations where frequent inspection of the injury site is necessary. However, they may not provide adequate stabilization for certain fractures, necessitating the use of casts or surgical intervention in more severe cases [25, 27]. Muscle atrophy is a common issue following immobilization, as reduced physical activity leads to decreased muscle mass and strength. This atrophy can complicate recovery, especially in older patients, and highlights the importance of early mobilization and physical therapy. Early intervention with physical therapy can significantly reduce recovery time and improve functional outcomes. Physical therapy postcast removal helps restore strength and flexibility, with tailored exercise programs addressing the specific needs of the patient [28, 29].

TENS offers a noninvasive method for pain relief. The device uses low-voltage electrical currents to alleviate pain by either blocking pain signals or increasing the release of endorphins. It is used for various conditions, including osteoarthritis and chronic pain. TENS is generally safe, though some patients may experience minor side effects [30]. It provides an excellent alternative to pharmaceutical pain management, particularly for patients with contraindications to medications.

#### 1.1.1. Ottawa Ankle Rules

**Figure 1:**
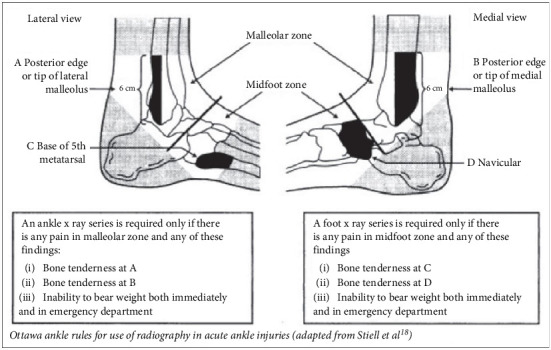
Ottawa Ankle Rules for the use of radiography in acute ankle injuries. This figure illustrates the Ottawa Ankle Rules, a clinical decision tool used to determine the need for radiographic imaging in patients with acute ankle and foot injuries. The lateral and medial views of the ankle are used to identify specific anatomical landmarks within two distinct zones: the malleolar zone and the midfoot zone. An ankle x-ray series is required only if the patient presents with pain in the malleolar zone and at least one of the following findings: (A) bone tenderness at the posterior edge or tip of the lateral malleolus and (B) bone tenderness at the posterior edge or tip of the medial malleolus or inability to bear weight both immediately and during evaluation in the emergency department. A foot x-ray series is required only if there is pain in the midfoot zone along with one or more of the following: (C) bone tenderness at the base of the fifth metatarsal and (D) bone tenderness at the navicular bone or inability to bear weight both immediately and in the emergency department. These guidelines are intended to reduce unnecessary imaging while ensuring clinically significant fractures are not missed (adapted from [33]).

The Ottawa Ankle Rules (Figure 1) provide a clinical guideline for determining the need for radiography in ankle injuries. Ottawa Ankle Rules were included as foundational guidelines for initial clinical assessment integrated directly into the expert system's diagnostic logic. By assessing bone tenderness and the ability to bear weight, these rules help reduce unnecessary x-rays, thus lowering healthcare costs and minimizing radiation exposure. The guidelines have proven effective in emergency settings, ensuring efficient and accurate diagnosis [31–33]. This evidence-based approach improves patient care by reducing the risk of unnecessary procedures and focusing on resources where they are most needed.

### 1.2. Technical Overview

#### 1.2.1. Knowledge-Based Systems

Knowledge-based systems are programs designed to solve complex problems using a structured set of knowledge and reasoning capabilities. They consist of three main subsystems: a knowledge base, a user interface, and an inference engine. The knowledge base organizes and displays facts about the world, while the inference engine uses logical rules, often in the form of IF-THEN statements, to make decisions or solve problems. This setup is typical in production systems, a type of artificial intelligence software utilized for expert systems, automated planning, and decision-making [34, 35].

The architecture of a knowledge-based system involves several components, including knowledge acquisition, an interface, a knowledge base, a working memory loader, an inference machine, and an explanation mechanism. Knowledge acquisition involves capturing and testing the knowledge base to ensure accuracy and consistency. The interface subsystem facilitates interaction between users and the system, allowing for the efficient construction and modification of the knowledge base. The knowledge base itself contains a facts base, production rules set, and ontology, forming the core of the system's decision-making capability. The memory loader initializes the system's state, while the inference machine applies rules to the current state to achieve goals. The explanation mechanism provides users with explanations of how decisions are made, ensuring transparency and trust in the system's outputs [34].

#### 1.2.2. 3D Modeling

3D modeling was employed to visualize and prototype the integration of the TENS device within the orthopedic cast, aiding in design validation. 3D modeling is the process of creating a three-dimensional representation of objects or surfaces by manipulating polygons, edges, and vertices in a simulated 3D space. This process begins with data collection and results in a virtual model that can be viewed and interacted with on a computer. It is widely used in fields like inspection, navigation, object identification, visualization, animation, and product design, particularly in CAD/CAM manufacturing and 3D printing [36].

3D modeling has become more accessible due to advancements in computer technology, allowing its use on low-cost computers. Noncontact technologies, such as those using light waves, are commonly employed in creating 3D models. Popular 3D modeling software includes AutoCAD, ZBrush, SketchUp, and Blender, which offer tools for designing both simple and complex models [37]. CAD systems, fundamental in creating detailed virtual models for physical 3D printing, provide cost-effective and efficient solutions for various engineering and architectural applications [38].

## 2. Literature Review

Orthopedic care has witnessed transformative advancements, from traditional casting methods to intelligent diagnostic tools and personalized treatment systems, all contributing to better patient outcomes and care efficiency.

Orthopedic casting has evolved significantly from primitive materials to modern synthetic options that enhance patient comfort and healing. Traditional plaster casts remain prevalent due to their cost-effectiveness and adaptability, while synthetic alternatives are often used for short-term or extremity cases [39]. Materials such as plaster of Paris, fiberglass, and thermoplastics each offer unique benefits and limitations, with known complications including pressure sores and deep vein thrombosis [39, 40]. To address these issues, innovations such as 3D-printed and foam-based casts like FlexiOH have been developed to improve ventilation and customization [40]. In clinical practice, serial casting continues to be effective for conditions like early-onset scoliosis, potentially delaying invasive surgical interventions [41].

Beyond materials, treatment efficacy in orthopedics can be enhanced through adjunct therapies like TENS. A meta-analysis demonstrated that TENS produces a medium-sized, statistically significant effect in managing central pain in patients with multiple sclerosis [42]. Mechanistically, TENS is thought to activate pain inhibition through the gate control theory and the release of endogenous opioids [19]. In orthopedic settings, TENS has shown effectiveness in managing acute and chronic musculoskeletal pain [43, 44]. Notably, when combined with therapeutic exercises, TENS significantly improved quadriceps activation in tibiofemoral osteoarthritis patients compared to exercise or placebo TENS alone [45]. However, literature reveals inconsistencies in TENS application protocols, particularly in acute hospital settings [46]. While some reviews are inconclusive due to methodological limitations, larger meta-analyses affirm TENS's positive outcomes for musculoskeletal and postoperative pain [44]. Given its safety, affordability, and patient acceptance, further standardization of TENS protocols is essential for maximizing its benefits across orthopedic conditions.

To further optimize orthopedic diagnosis and treatment planning, knowledge-based expert systems offer valuable support. These systems are composed of a knowledge base and an inference engine and often include interfaces for explanation, acquisition, and user interaction [47, 48]. They use diverse representation schemes such as predicate logic, frames, and production rules to encode expert knowledge and make intelligent decisions [49, 50]. The development of such systems involves the integration of both factual and heuristic knowledge from domain experts [47]. Advanced capabilities like nonmonotonic and temporal reasoning broaden their application scope [49], making them suitable for complex clinical scenarios.

Recent applications of expert systems and AI in orthopedics and orthodontics demonstrate considerable promise. Case-based expert systems have been applied in orthodontics for treatment planning, while predictive models using tree-based data mining are used in pediatric orthopedics to identify conditions like clubfoot and rotational abnormalities [51, 52]. AI tools now support automated tasks such as cephalometric landmark detection, bone age estimation, and orthognathic surgery planning [53]. These tools enhance diagnostic accuracy and reduce clinical burden, especially for less experienced practitioners. AI techniques in medicine encompass rule-based reasoning, neural networks, case-based reasoning, and fuzzy logic, often used in hybrid forms to boost performance [54]. These technologies are applied to image interpretation, clinical decision-making, and risk prediction in orthopedic care [55–57]. Although concerns remain regarding data privacy, algorithmic bias, and legal constraints [55], the potential of AI—especially in complex orthopedic domains such as the spine, knee, and hip—is evident, with expanding roles in surgical robotics, remote monitoring, and personalized care [56, 57].

Complementing these AI applications, 3D simulations and prototyping are increasingly shaping personalized medical device development. These technologies accelerate the design cycles, reduce production costs, and allow for highly customized solutions for implants and prosthetics [58]. Rapid prototyping and 3D printing facilitate iterative testing and stakeholder collaboration during early development stages [59, 60]. Computational techniques such as finite element analysis and fluid dynamics are used to simulate device performance before production [59, 61]. Nonetheless, challenges persist, including regulatory barriers and limitations in 3D printing resolution, necessitating close collaboration between engineers and clinicians [58, 61].

Several digital applications have emerged to support orthopedic diagnosis and treatment, though most lack full integration of AI and smart device capabilities. For instance, MoonlightOrtho is a telehealth platform offering musculoskeletal care via asynchronous video assessments yet lacks AI-based diagnostics and physical rehabilitation integration [62]. Implant Identifier leverages AI and image processing to match implants to radiographs but does not provide diagnostic or treatment recommendations [63]. OrthoLive facilitates secure telemedicine consultations but is limited in AI capabilities and imaging support [64]. OsteoDetect uses AI to detect wrist fractures from x-rays but does not support treatment recommendations or smart device integration [65]. In contrast, the proposed system integrates knowledge-based reasoning, image processing, feedback mechanisms, diagnosis, treatment recommendations, and device-based therapy, offering a more holistic solution as summarized in Table 1.

In conclusion, while current orthopedic technologies offer significant advancements in casting materials, adjunct therapies like TENS, and AI-based support systems, there remains a gap in integrating these components into a single, comprehensive system. The proposed solution aims to bridge this gap by uniting modern casting, TENS therapy, AI-based diagnostics, and smart rehabilitation devices. Such integration is vital for delivering patient-centered care that is effective, efficient, and personalized—ushering in a new era of orthopedic healthcare innovation.

## 3. Methodology

### 3.1. System Overview

This study proposes a solution to enhance the diagnosis, follow-up, and treatment of orthopedic injuries. The solution consists of two main components: (i) an application (knowledge-based expert system) for patients and clinicians and (ii) prototype devices for orthotics and rehabilitation. The first component is an application designed to serve both patients and clinicians. This application is aimed at providing an initial diagnosis of injuries. Patients use the application to diagnose their injuries by answering a series of questions. The expert system processes these responses and displays diagnostic results, which include an initial diagnosis of the injury and treatment recommendations.

Clinicians can also use the application to manage patient care. They can add patients who seek treatment, supervise recovery plans stored in the system for various injuries, and edit these plans as needed before sending them to patients. The application facilitates remote follow-up from the onset of the injury, enabling continuous monitoring and management of the patient's condition.

**Figure 2:**
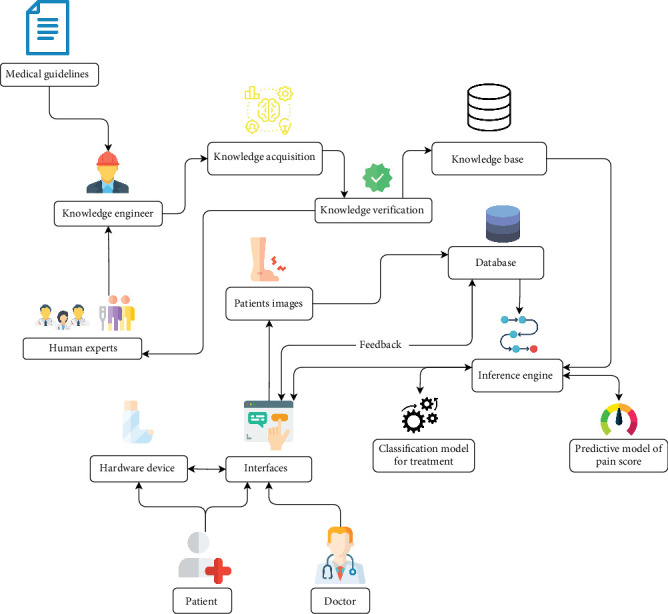
Framework of the knowledge-based expert system for orthopedic diagnosis and treatment. This figure illustrates the comprehensive framework of the TeleOrtho system designed for diagnosing and managing ankle injuries. The process begins with knowledge acquisition from medical guidelines and human experts, facilitated by knowledge engineers. Verified knowledge is structured into a knowledge base that supports the inference engine in generating diagnoses and treatment recommendations. Patient data, including images, is stored in a secure database. The inference engine utilizes classification rules and a predictive model to determine treatment paths and pain scores. The system features user interfaces for both patients and doctors, allowing for data input, communication, and therapy supervision. The hardware device—a smart orthopedic cast with integrated TENS—delivers personalized therapy. This architecture ensures accurate, real-time, and patient-specific orthopedic care through AI-driven decision support.

The second component is a prototype device designed for orthotics and injury rehabilitation. This device is an orthopedic cast integrated with a TENS machine. The TENS machine delivers electrical pulses to relieve pain and maintain muscle activity. The device is connected to the application, allowing specialists to control the level of electrical pulses remotely. This dual-component system aims to streamline the entire process of diagnosing, treating, and rehabilitating orthopedic injuries, ensuring timely and effective patient care. The integration of the application and the prototype device represents a significant advancement in orthopedic treatment, leveraging technology to improve patient outcomes and clinician efficiency.  Figure 2 depicts the various components involved in the knowledge-based expert system. It includes medical guidelines, knowledge acquisition, and verification processes, forming a knowledge base maintained by knowledge engineers and human experts. Patient data and images are stored in a database, and an inference engine uses classification and predictive models to provide treatment recommendations and pain score predictions. Interfaces allow doctors and patients to interact with the system, while hardware devices facilitate the implementation of recommended treatments.

**Figure 3:**
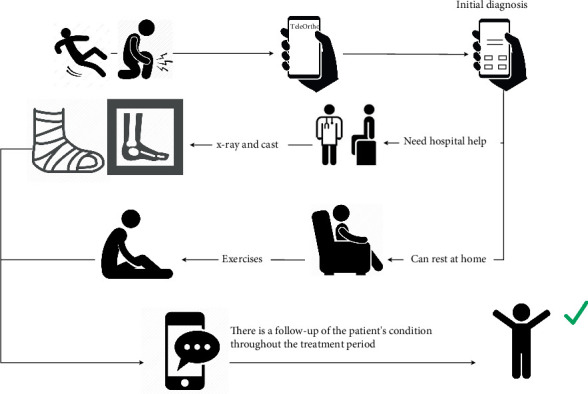
Decision flow for initial diagnosis and treatment recommendation in the TeleOrtho system. This figure illustrates the patient's journey after an injury using the TeleOrtho application. The process begins with the patient answering diagnostic questions via the app. Based on the responses, the system provides one of two recommendations: (a) visit clinician—the patient is advised to seek hospital care for procedures such as x-ray imaging, casting, and supervised TENS therapy; or (b) home care—the patient is instructed to rest and perform prescribed exercises at home. In both cases, continuous remote follow-up is conducted through the application to ensure proper recovery and patient satisfaction.

When a person is injured, they use the application to make an initial diagnosis by answering questions that will provide diagnostic results and recommendations. The recommendations will fall into one of two categories, as shown in Figure 3:

1. Visit clinician: In this case, the patient is advised to visit a clinician who will perform necessary procedures such as x-rays, applying a cast, and administering TENS therapy, which the clinician controls.
2. Home care: In this scenario, the system recommends that the patient rest at home and perform specific exercises. The patient will receive remote follow-up through the application during the treatment period.

In both scenarios, there will be continuous remote follow-up throughout the treatment period until its conclusion, ensuring comprehensive care and monitoring facilitated by the application.

### 3.2. Knowledge Acquisition

In the TeleOrtho system, knowledge acquisition involves collecting and verifying information regarding ankle injuries, specifically ankle fractures and ankle sprains. This process includes gathering symptoms, risk factors, diagnostic recommendations, and recovery plans as outlined in medical guidelines. These guidelines are then validated by orthopedic specialists to ensure accuracy and reliability, ensuring that the system's knowledge base is built on a solid foundation of verified medical information.

We used two methods to gather knowledge: expert input and medical guidelines. First, we utilized an expert questionnaire to facilitate access to the insights of orthotists and physiotherapists. This questionnaire was designed using Google Forms, with most questions being closed-ended (Table 2). The survey is aimed at gathering information about orthopedic injuries and reinforcing the facts obtained through interviews.

Additionally, the knowledge base IF-THEN rules were developed using explicit clinical guidelines, specifically Ottawa Ankle Rules and validated by orthopedic specialists (see Supporting Information S1) [33, 66].

#### 3.2.1. The Diagnostic Process

The diagnostic process in our expert system is based on a structured set of questions derived from the Ottawa Ankle Rules [31–33]. These questions help determine the nature and severity of the injury by assessing symptoms and identifying contraindications to the use of TENS therapy. The output of this process is an initial diagnosis report, which includes potential injuries, associated symptoms, and recommended actions.

#### 3.2.2. The Suggested Recovery Plan

Upon receiving an initial diagnosis, patients are presented with a proposed recovery plan [33, 66], including specific exercises. This plan is reviewed and potentially modified by a specialist before being finalized and made available to the patient. The recovery plans for ankle fractures (Weber A [67] and B [68]) include detailed timelines and exercises to ensure optimal healing and recovery. More details are included in Supporting Information S1.

Ankle fracture types and recovery plans:

• Weber A: This type of fracture is an avulsion injury below the talus level. It typically requires 6 weeks to heal, though pain and swelling may persist for up to 6 months. Recovery involves using a cast and following specific rehabilitation exercises.
• Weber B: This fracture type usually involves a spiral injury at the ankle joint level, potentially affecting the medial malleolus and ligaments. The recovery process is like Weber A, with additional considerations for the injury's complexity.

#### 3.2.3. TENS Placement Guide

TENS units are used to deliver electrical impulses that stimulate the body's natural pain-relieving mechanisms. The device uses two pads placed over or near the affected area to emit ultrasound signals, which help release opioid-like chemicals in the body [30, 69]. Proper electrode placement is essential for effective TENS therapy. Patients should place the electrodes near the area of pain, following the illustrated guides provided by the TeleOrtho application. To ensure proper usage, the skin must be cleaned and dried before placing the electrodes. The electrodes should be attached, with adhesive used if necessary.

### 3.3. Knowledge Base

In the TeleOrtho system, the knowledge base comprises a structured set of facts, rules, and ontologies related to diagnosing and treating ankle injuries. This knowledge base is developed from the latest medical guidelines and expert opinions, ensuring accuracy and reliability. According to the production model in expert systems, problems are solved by applying knowledge (expressed as production rules) to specific issues represented by problem-specific information. This knowledge is the sum of what is currently known and is typically represented in AI as IF-THEN rules, which relate provided rules or facts in the IF section to some action in the THEN part. Any rule has two parts: the IF part, known as the antecedent (premise or condition), and the THEN part, known as the conclusion or action [70]. No training dataset was utilized, as the knowledge base was manually defined by experts based on medical guidelines and literature rather than through data-driven training methods.

### 3.4. Knowledge Engineers and Users

The development and maintenance of the TeleOrtho system involves a collaborative effort between knowledge engineers, human experts (such as orthopedic specialists), and users (patients and doctors). Knowledge engineers are responsible for encoding expert knowledge into the system, while human experts provide the necessary medical knowledge and validate the system's outputs. Users interact with the system through a user-friendly interface, ensuring that the information provided is accessible and understandable.

### 3.5. Database Design

The system's database serves as a repository for comprehensive medical information, patient records, and system-generated outputs. The inference engine utilizes this data, leveraging the rules defined in the knowledge base to diagnose conditions and suggest appropriate treatments.

### 3.6. Classification Model for Treatment

The classification model is primarily a rule-based expert system employing IF-THEN rules derived from validated medical guidelines. Future iterations may incorporate machine learning algorithms for automated knowledge extraction and enhanced predictive capabilities. The classification model within the TeleOrtho system is designed to categorize injuries and recommend appropriate treatments. It uses data from the knowledge base to precisely identify the type and severity of an injury, differentiating between various conditions such as ankle fractures, ankle sprains, the absence of injury, appropriate use of a cast, and situations where cast use is prohibited. This model employs forward chaining [50, 70], a method in which the system begins with the available data and applies rules to infer conclusions, thereby providing tailored treatment recommendations for each patient. No mathematical equations or machine learning algorithms were used since the system is fully based on manually defined IF-THEN rules. This approach ensures that patients receive individualized treatment plans, enhancing the precision and effectiveness of medical interventions.

### 3.7. Predictive Model of Pain Score

The predictive model of pain score in the TeleOrtho system is designed to estimate the patient's pain level, particularly concerning and adjustment of TENS therapy. This model dynamically adjusts based on real-time feedback from the patient and clinician inputs. Patients can report their current pain levels through a series of questions, allowing the system to update the pain score accordingly. The system also enables patients to control their TENS therapy, including the option to stop the treatment if necessary.

Clinicians play a crucial role in this model by monitoring the reported pain scores and TENS usage. They can use this data to refine the treatment plan, ensuring that the therapy remains effective and appropriate for the patient's condition. This collaborative approach between patients and clinicians ensures a personalized and adaptive pain management strategy, leveraging real-time data to optimize the patient's comfort and treatment outcomes.

### 3.8. User Interfaces

The TeleOrtho system features an intuitive user interface that facilitates easy interaction for both patients and clinicians. Patients can use the interface to input symptoms, view diagnostic results, and follow recovery plans. Clinicians can access patient records, modify treatment plans, and communicate with patients. The interface is designed to be accessible and user-friendly, ensuring that users can easily navigate the system and access necessary information.

### 3.9. Hardware Device (3D Modeling)

The hardware component of the TeleOrtho system includes a 3D model that simulates the prototype for the treatment of ankle injuries. This model helps in visualizing the application of the treatment, such as the placement of a cast and the use of a TENS machine. The 3D model provides a realistic representation of the treatment process, helping both patients and clinicians understand the application and expected outcomes.

### 3.10. Implementation

The implementation of the TeleOrtho system involves a strategic integration of several advanced technologies to ensure efficient and reliable performance. Dart [71] and Flutter [72] are utilized to develop cross-platform applications, enabling the creation of natively compiled, visually appealing apps from a single codebase. This setup is complemented by Firebase [73], a robust Backend-as-a-Service solution, which provides essential backend functionalities such as user authentication and real-time databases. Python [74] is employed for the development of the expert system, leveraging its simplicity and efficiency in handling complex machine learning and AI processes. SQLite [75] serves as the embedded relational database, offering a lightweight yet powerful solution for data management, while PyCharm Integrated Development Environment (IDE) [76] streamlines Python development with its extensive features and support for various operating systems.

For hardware simulation, the Arduino IDE [77] and Autodesk Tinkercad [78] are employed. The Arduino IDE simplifies the development of interactive hardware projects, allowing for easy code writing and uploading to the Arduino microcontroller. Tinkercad provides a suite of tools for 3D design and electronic circuit simulation, including an Arduino controller emulator, making it ideal for prototyping and testing hardware components.

### 3.11. Knowledge-Based Expert System Testing

#### 3.11.1. Static Testing

Static testing involves reviewing the system's code and documentation without executing the software. It focuses on detecting software flaws and emphasizing quality throughout the software lifecycle. The primary technique used was a walkthrough, where team members reviewed the system's components, such as knowledge acquisition, verification, functional and nonfunctional requirements, and expert system rules.

#### 3.11.2. Dynamic Testing

Dynamic testing was conducted in a real-world environment to verify that the system met user needs and specifications by testing its functional behavior. This testing included both functional and nonfunctional requirements. Functional testing ensured that the system's components, such as the application and expert system, worked as expected. For instance, unit tests were performed on various components like sign-up, log in, and initial diagnosis, confirming that they functioned correctly. Integration testing further verified that all system components worked together seamlessly, ensuring data transmission between them.

#### 3.11.3. Integration Testing

Integration testing was carried out to ensure that the various components of the TeleOrtho system worked together cohesively. This level of testing aimed to identify flaws in how different software modules interacted when combined. The process involved checking data transmission between units, verifying that the system could perform tasks like controlling TENS intensity and sending emergency requests without issues.

#### 3.11.4. Case Study

This study did not involve real patients. The diagnostic process and system validation were performed using simulated data based on published case studies. This involved testing the system's ability to diagnose injuries and providing treatment recommendations based on user inputs. Therefore, ethical approval was not required.

## 4. Results

### 4.1. Knowledge-Based

In the TeleOrtho system, the knowledge base currently comprises five main rules (Table 3) with nine specific subconditions clearly outlined in Table 4.

### 4.2. User Interface

**Figure 4:**
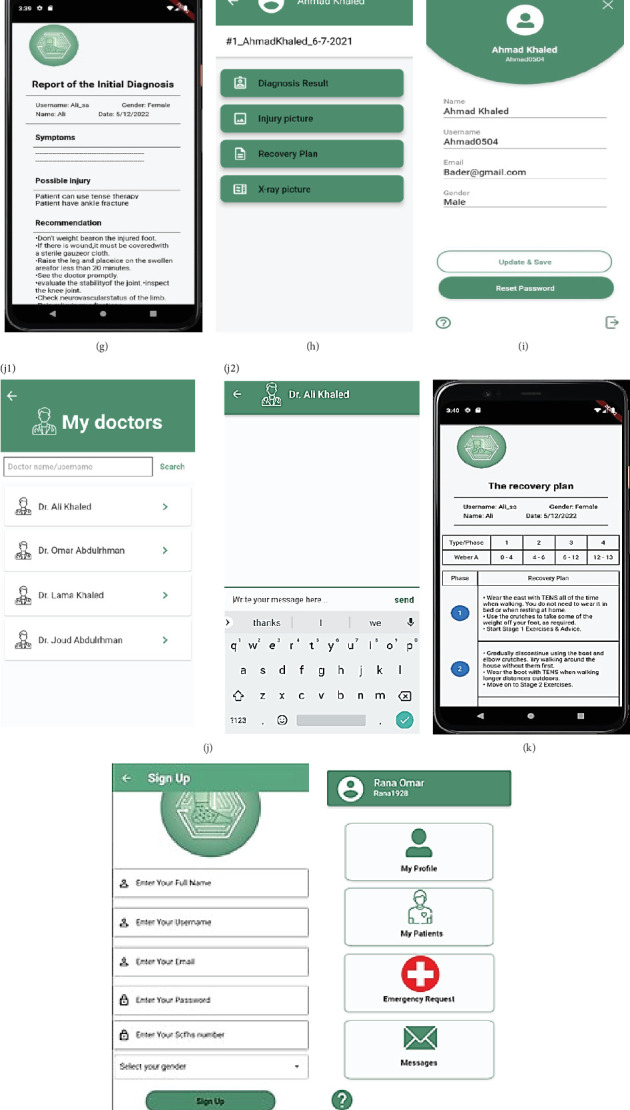
(a–p) Screenshots of the TeleOrtho application's user interfaces.

**Figure 5:**
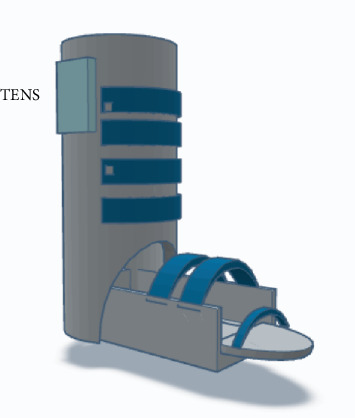
3D modeling for the TeleOrtho Cast.

The implementation of the TeleOrtho application features distinct interfaces for two user types: patients and clinicians. This section provides an overview of the general interfaces shared by both user groups and specific interfaces tailored to the unique needs of patients and clinicians. The user interfaces of the TeleOrtho application are detailed in Table 5 and visually represented in Figures 4 and 5. These elements illustrate the comprehensive design and functionality provided to both patients and clinicians, facilitating a seamless user experience. Table 5 summarizes the various user interfaces within the TeleOrtho application, categorized into general interfaces, patient-specific interfaces, and clinician-specific interfaces. Each interface is described based on its primary function and user interaction.  Figure 4 provides a visual overview of the user interfaces in the TeleOrtho application, showcasing key screens for both patients and clinicians. The subfigures (Figures 4, 4, 4, 4, 4, 4, 4, 4, 4, 4 (j-1 and j-2), 4, 4, 4, 4, 4, and 4 (p-1 to p-4)) correspond to different sections of the application, highlighting the specific functionalities available to each user group.

### 4.3. Database Implementation

The relational database schema, outlined in Supporting Information S2, is designed to efficiently organize and store data related to patient information, medical history, diagnostic results, and treatment plans. This structured approach ensures that all data is accessible and can be retrieved quickly, supporting the system's real-time decision-making capabilities.

The database's architecture is carefully designed to accommodate the system's requirements, including user authentication, data integrity, and secure storage of sensitive medical information. Tables within the database are normalized to minimize redundancy and maintain data consistency. The scheme includes tables for user credentials, patient records, clinician details, diagnostic images, and treatment plans, among others. Each table is linked by unique identifiers, ensuring relational integrity and enabling complex queries across the system.

Additionally, the flowchart diagram of patient's authorities (Supporting Information S3) and the data flow diagram (DFD) (Supporting Information S4) visually depict the data management processes and user interactions within the system. These diagrams illustrate how data flows between different modules, from user input to the storage of diagnostic results and treatment recommendations. They also highlight the security measures in place to protect patient data, including access control mechanisms and encryption protocols.

### 4.4. Hardware Device (3D Modeling)

The TeleOrtho system includes a 3D-modeled orthopedic support device, as illustrated in Figure 5. This device is designed for ankle stabilization during recovery, featuring a lightweight structure with adjustable straps for a customized fit. The model ensures straightforward application and removal, promoting patient independence in managing their care. Integrated with the TENS therapy system, this hardware component provides both physical support and therapeutic electrical stimulation, enhancing the overall functionality and effectiveness of the TeleOrtho system.

### 4.5. Knowledge-Based Expert System Testing Results

To assess the performance and usability of the proposed TeleOrtho system, a series of evaluations were conducted involving simulated case scenarios, static and dynamic tests, and initial user feedback. The evaluation focused on three main areas: system accuracy, response time, and user experience.

#### 4.5.1. Static Testing

Static testing, which includes code reviews and walkthroughs, ensured that the system's code quality and adherence to design specifications were maintained. The walkthrough sessions highlighted a few potential issues, such as ambiguous button placement, which were addressed promptly. The static testing confirmed that the system met the defined requirements and standards, ensuring a robust foundation for the dynamic testing phase.

#### 4.5.2. Dynamic Testing

Dynamic testing focuses on evaluating the system's performance under real-world conditions. This phase included unit testing, where individual components, such as the expert system's logic and the application interfaces, were tested in isolation. The results showed that all components functioned as expected. Specifically, the system accurately performed initial diagnoses and subsequent recommendations based on user inputs. The dynamic tests also covered usability aspects, confirming that the interface was intuitive and user-friendly for both clinicians and patients.

#### 4.5.3. Integration Testing

Integration testing was critical in ensuring that different modules of the system worked together seamlessly. This phase tested the interactions between the application, expert system, and hardware components, such as the TENS device. The system successfully integrated these components, facilitating smooth data flow and consistent performance. Notably, the expert system's rules were correctly applied to user data, and the hardware responded appropriately to commands from the application.

#### 4.5.4. Case Study Analysis

The knowledge-based expert system was tested using two published orthopedic case scenarios. In both cases, the system provided diagnostic results and treatment recommendations that aligned with those of licensed clinicians, confirming the correctness of the embedded rules and reasoning logic. Two case studies demonstrated the system's practical applications. These included scenarios: diagnosing ankle injuries (fracture [79] and sprain [80]). The system's performance in these case studies was consistent with the expected outcomes, confirming its reliability and effectiveness. The case studies highlighted the system's capability to provide accurate diagnoses and appropriate treatment recommendations.  Table 6 presents the application of the knowledge-based system to classify two patients based on their responses to the provided questions, as derived from published case studies. The classification outcome reflects the diagnosis for each patient according to the applied rules. Due to the knowledge-based nature of the system, validation focused on case-study–based verification (Table 6) rather than traditional model accuracy evaluation metrics. Therefore, performance metrics such as sensitivity, specificity, or accuracy typically associated with data-driven AI models were not applicable due to the rule-based nature of the system.

Performance testing measured the system's average response time from the submission of patient symptoms to the delivery of diagnostic output. The system demonstrated an average response time of 1.8 s, ensuring prompt feedback suitable for real-time use.

#### 4.5.5. Usability and User Satisfaction

An initial usability test was conducted using the System Usability Scale (SUS). Two participants completed a series of core tasks, including account creation, initial diagnosis, and report viewing (Supporting Information S5). The average SUS score obtained was 85.3/100, indicating an important level of satisfaction and ease of use.

In addition to the SUS score, the following human factors metrics were recorded:

• Task completion rate: 100% across all tasks
• Average task operation time: 6–10 s per function
• Error rate: 0% during all test interactions

Participants noted that the application was user-friendly and that the step-by-step diagnostic process was easy to follow. Suggestions for improvement included adding a help button, tooltips for input fields, and visual enhancements for diagnosis result displays. These recommendations have been documented for future development iterations.

## 5. Discussion

The TeleOrtho system offers several advantages. It provides accurate and efficient initial diagnoses, helping reduce the need for in-person consultations. The system's integration of real-time patient feedback, especially in managing pain through TENS therapy, allows for a more personalized treatment approach. The use of a comprehensive knowledge base ensures that the recommendations are based on up-to-date medical guidelines and expert opinions.

The TeleOrtho system contributes significantly to telemedicine and orthopedic care. It provides a framework for remote diagnosis and treatment, potentially reducing healthcare costs and improving patient accessibility. The system's comprehensive database and use of artificial intelligence in diagnostics and treatment planning represent significant advancements in healthcare technology. The project's outcomes can be further expanded to include more complex orthopedic conditions, enhancing its utility in various medical fields.

Despite its strengths, the system has limitations. One of the main challenges is the reliance on patient self-reporting, which can sometimes lead to inaccurate data. The system also lacks integration with advanced imaging technologies, such as MRI or CT scans, which are crucial for diagnosing complex injuries. Additionally, the effectiveness of TENS therapy is still a topic of debate in the medical community, which may limit its applicability to all patients. Indeed, the effectiveness of TENS for chronic pain management remains controversial. While some studies support its use, others refute its clinical efficacy [19]. A comprehensive review highlights the need for large multicentered randomized controlled trials (RCTs) using enriched enrollment designs to resolve the efficacy impasse [81]. A systematic review of RCTs for chronic low-back pain found conflicting evidence regarding TENS effectiveness in pain reduction and functional improvement [82]. Methodological limitations, including clinical heterogeneity and small sample sizes, have hindered definitive conclusions. Despite these uncertainties, TENS remains widely used due to its noninvasive nature and few contraindications [19]. Future research should focus on optimizing TENS techniques, treatment schedules, and integration into existing pain management strategies to better understand its potential benefits [81].

In addition, future work on the TeleOrtho system will focus on several key areas to enhance its capabilities and applicability. Firstly, integrating advanced imaging technologies, such as MRI and CT scans, into the diagnostic process will improve the accuracy and scope of the system, allowing for more comprehensive assessments of complex injuries. Additionally, expanding the knowledge base to include a broader range of orthopedic conditions and treatments will increase the system's versatility. Enhancing the machine learning models to incorporate more patient data, including demographics and medical history, will improve the predictive accuracy of pain scores and treatment outcomes. In addition, While the results are promising, the current evaluation involved a small sample size (*n* = 2 case studies), which limits the generalizability of the findings, future research will include comprehensive validation studies involving clinical trials and comparative evaluations to provide robust evidence supporting the system's effectiveness. These comparative evaluations will include control groups receiving non-TENS treatments to rigorously assess clinical effectiveness. Finally, developing a more robust patient interface with features for better self-reporting and real-time communication with healthcare providers will enhance the overall user experience and engagement. These advancements will not only improve the system's diagnostic and treatment capabilities but also broaden its application in telemedicine and remote healthcare services.

## 6. Conclusion

In conclusion, the knowledge-based expert system designed for orthopedic diagnosis and treatment, particularly for ankle injuries, has shown significant potential in providing accurate and personalized healthcare solutions. The system's ability to utilize medical guidelines and expert input to formulate IF-THEN rules has resulted in an effective tool for diagnosing conditions such as ankle fractures and sprains. This approach not only ensures accurate diagnoses but also helps in devising comprehensive treatment plans, including the appropriate use of TENS therapy. The system's practical application demonstrated the potential for reducing misdiagnosis and optimizing recovery outcomes, thus improving patient care.

However, the study also highlighted some limitations. The reliance on predefined rules may not capture the full complexity of certain cases, and the system's accuracy depends on the quality and comprehensiveness of the input data. In addition, the TENS therapy efficacy varies and may be limited for certain patient groups, particularly those with neuropathic pain or contraindications such as epilepsy or pregnancy. Furthermore, while the system's recommendations align well with standard medical guidelines, further research is needed to refine the decision-making process and integrate more nuanced medical conditions. Future developments should focus on enhancing the system's adaptability, incorporating real-time data analysis, and expanding its scope to cover a broader range of orthopedic issues, thereby making it a versatile tool in the field of telemedicine and remote patient management. Future clinical studies will seek Institutional Review Board (IRB) approval to validate the system with real patient data.

## Figures and Tables

**Table 1:** Comparison between existing orthopedic applications and the proposed system.

**Software**	**Knowledge-based**	**Image processing**	**Feedback**	**Initial diagnosis**	**Treatment recommendation**	**Smart device**
MoonlightOrtho [62]				√	√	√
Implant Identifier [63]				√	√	√
OrthoLive [64]	√	√				
OsteoDetect [65]	√	√		√		
Proposed project	√	√	√	√	√	√

**Table 2:** Questionnaire questions and purposes.

**Question**	**Purpose**
What part of the body is most often injured and needs physiotherapy?	Identify the most frequently injured body part requiring physiotherapy.
What are the most common injuries that require physiotherapy after the splint/cast?	Determine the common injuries needing physiotherapy postsplint/cast.
Do electric stimulation pulses help in physiotherapy or in treating muscle inactivity?	Assess the effectiveness of TENS therapy in physiotherapy and treating muscle inactivity.
Do you prefer having an application that helps the clinician monitor the patient's condition remotely during the treatment period?	Evaluate the demand and utility of a remote monitoring application for patients.
Medically, is it possible to treat the injury and maintain muscle activity during one period?	Explore the feasibility of simultaneous injury treatment and muscle maintenance.
Please write the reason for the answer to the previous question.	Gather expert opinions on the simultaneous treatment of injuries and muscle activity maintenance.

**Table 3:** The medical knowledge represented as IF-THEN rules.

**Rule**	**IF conditions**	**THEN outcome**
Rule 1: Ankle fracture	Case 1: (no in all G1) AND (yes in any of G2) AND (yes in any of G3-A or G3-B)	High possibility of ankle fracture
	Case 2: (yes in any of G1) AND (yes in any of G3-A or G3-B)	High possibility of ankle fracture

Rule 2: Ankle sprain	Case 1: (no in all G1) AND (no in all G2) AND (yes in any of G4-A or G4-B)	High possibility of ankle sprain
	Case 2: (yes in any of G1) AND (no in all G3-A) AND (yes in any of G4-A or G4-B)	High possibility of ankle sprain
	Case 3: (no in all G1) AND (yes in any of G2) AND (no in all G3-A) AND (yes in any of G4-A or G4-B)	High possibility of ankle sprain

Rule 3: No injury	Case 1: (no in all G1) AND (no in all G2) AND (no in all G4-A)	The patient does not have an injury
	Case 2: (yes in any of G1) AND (no in all G3-A) AND (no in all G4-A)	The patient does not have an injury
	Case 3: (no in all G1) AND (yes in any of G2) AND (no in all G3-A) AND (no in all G4-A)	The patient does not have an injury

Rule 4: Cast use	Case 1: (no in all G5)	The patient can use the cast

Rule 5: Cast prohibition	Case 1: (yes in any of G5)	The patient cannot use the cast

**Table 4:** Comprehensive question set for diagnostic and treatment assessment.

**Group**	**Questions**
G1: Contraindications of Ankle Ottawa Rules	Q1: Are you less than 18 years old? Q2: Do you have intoxication? Q3: Do you have multiple painful injuries? Q4: Are you pregnant? (only female) Q5: Do you have a head injury? Q6: Are you suffering from any of these conditions? Abnormal reflexes; inability to speak; decreased sensation; loss of balance; mental function problems, such as memory loss; vision changes; walking problems; or weakness of the arms or legs.

G2: Ankle Ottawa Rules	Q1: Place your fingers along the back and sides of your ankle. Apply gentle pressure to these spots. Check if you feel any pain or tenderness. This step helps determine if there may be a fracture in the ankle. Q2: Place your fingers around your midfoot and near the base of your toes. Apply pressure and observe if you feel any pain. This action is crucial for identifying potential fractures in these regions. Q3: Are you able to balance when you walk four steps?

G3-A: Ankle fracture	Q1: Do you have any pain? Q2: Do you have joint swelling? Q3: Do you have a deformity of the ankle?

G3-B: Ankle fracture	Q4: Do you have a discoloration of the skin? Q5: Do you have an absent pulse? Q6: Do you have osteoporosis? Q7: Do you smoke? Q8: To evaluate your BMI, enter the weight in kilograms and the height in centimeters. (You need to compute the BMI; if the result is elevated BMI, then there is a possibility of an ankle fracture.) Q9: Do you have ecchymosis?

G4: Ankle sprain	Q1: Did you twist your ankle? Q2: Do you have any swellings accompanied by pain? Q3: Do you have bruising?

G4-B: Ankle sprain	Q1: do you have discoloration in the skin? Q2: Do you have difficulty and pain while walking? Q3: Do you have stiffness in your ankle? Q4: To evaluate your BMI, enter the weight in kilograms and the height in centimeters. (You need to compute the BMI; if the result is elevated BMI, then there is a possibility of an ankle fracture.)

G5: TENS therapy	Q1: Do you have heart disease? Q2: Are you pregnant? (only female) Q3: Do you have seizures? Q4: Do you have convulsions? Q5: Do you have lymphedema? Q6: Do you have blood clots? Q7: Do you have epilepsy?

**Table 5:** Summary of user interfaces for the TeleOrtho application.

	**Interface name**	**Description**
General interface	(a) Welcome page	Initial page with buttons for patient and clinician logins or account creation.
	(b) Sign-in page	Allow users to log in by entering their email and password.
	(c) Reset the password page	Enables users to reset their password by validating their email.

Patient interfaces	(d) Patient sign-up page	Allow patients to create an account by providing personal information.
	(e) Patient main page	The central hub for patients, providing access to TENS control, condition summary, initial diagnosis, reports, profile, and messaging.
	(f) Initial diagnosis page	Facilitates the diagnostic process by allowing patients to upload injury pictures and answer diagnostic questions.
	(g) Diagnosis result page	Displays the results of the initial diagnosis, including injury, symptoms, and treatment recommendations.
	(h) My reports page	Enables patients to review their reports, which include diagnosis results, injury pictures, recovery plans, and x-ray images.
	(i) My profile page	Allow patients to view and edit their personal information and change their passwords.
	(j) Message page	Facilitates communication between patients and clinicians, with the ability to delete messages.
	(k) Recovery plan page	Displays the clinician-approved recovery plan for patients to follow.

Clinician interfaces	(l) Clinician sign-up page	Allow clinicians to create an account by providing personal information.
	(m) Clinician main page	The central hub for clinicians provides access to profiles, patient management, emergency requests, and messaging.
	(n) My patient pages	Enables clinicians to add new patients, view patient reports, and control TENS therapy intensity.
	(o) Emergency request page	Receives notifications when patients stop TENS therapy, enhancing follow-up processes.
	(p) Upload x-ray pages	Allow clinicians to upload x-ray images to patient reports.

**Table 6:** Patient classification table.

**Question group**	**Questions**	**Patient 1 (79) answer**	**Patient 2 (80) answer**
G1: Contraindications of Ankle Ottawa Rules	Q1: Are you less than 18 years old?	No	No
	Q2: Do you have intoxication?	No	No
	Q3: Do you have multiple painful injuries?	No	No
	Q4: Are you pregnant? (only female)	No	No
	Q5: Do you have a head injury?	No	No
	Q6: Are you suffering from any of these conditions? (Abnormal reflexes, etc.)	No	Yes

G2: Ankle Ottawa Rules	Q1: Place your fingers along the back and sides of your ankle. Apply gentle pressure to these spots. Check if you feel any pain or tenderness.	Yes	No
	Q2: Place your fingers around your midfoot and near the base of your toes. Apply pressure and observe any pain.	Yes	No
	Q3: Are you able to balance when you walk four steps?	No	Yes

G3-A: Ankle fracture	Q1: Do you have any pain?	Yes	Yes
	Q2: Do you have joint swelling?	Yes	No
	Q3: Do you have a deformity of the ankle?	Yes	No

G3-B: Ankle fracture	Q4: Do you have a discoloration of the skin?	Yes	No
	Q5: Do you have an absent pulse?	No	No
	Q6: Do you have osteoporosis?	Yes	No
	Q7: Do you smoke?	No	No
	Q8: To evaluate your BMI, enter the weight in kilograms and the height in centimeters.	Not specified	Not specified
	Q9: Do you have ecchymosis?	Yes	No

G4: Ankle sprain	Q1: Did you twist your ankle?	No answer	Yes
	Q2: Do you have any swellings accompanied by pain?		Yes
	Q3: Do you have bruising?		Yes

G4-B: Ankle sprain	Q1: Do you have discoloration in the skin?	No answer	Yes
	Q2: Do you have difficulty and pain while walking?		Yes
	Q3: Do you have stiffness in your ankle?		Yes
	Q4: To evaluate your BMI, enter the weight in kilograms and the height in centimeters.		Not specified

Applied rule		Rule 1: Case 1	Rule 2: Case 1

Classification outcome		High possibility of ankle fracture	High possibility of ankle sprain

## Data Availability

This study did not generate or analyze any new data. However, all relevant knowledge and supporting information are available in the supporting information provided with this manuscript.

## Supporting Information

Supporting Information Additional supporting information can be found online in the Supporting Information section. S1: Medical guidelines. S2: Relational database schema. S3: Flowchart diagram of patient's authorities. S4: Data flow diagram. S5: Usability and user satisfaction.
