# Supplementary material for: Knowledge-Based Expert System for Orthopedic Injury Management With Integrated Transcutaneous Electrical Nerve Simulation Therapy
Source: Int J Telemed Appl. 2025 Jun 23;2025:5878245. doi: 10.1155/ijta/5878245 (PMC12208762; doi:10.1155/ijta/5878245)

Knowledge-based Expert System for Orthopedic Injury Management with Integrated Transcutaneous Electrical Nerve Simulation Therapy

Haneen Banjar^1,2,3,4^, Shahad Almalki^1^, Lama Almehmadi^1^, Amjad Alshahrani^1^, Ali Chaudhary^6^ and Reda Ghoname^5^

^1^ Computer Science Department, Faculty of Computing and Information Technology, King Abdulaziz University, Jeddah 21589, Saudi Arabia; [hrbanjar@kau.edu.sa](mailto:hrbanjar@kau.edu.sa), [salmalki0604@stu.kau.edu.sa](mailto:salmalki0604@stu.kau.edu.sa), Lalmihmadi@stu.kau.edu.sa, and aalshahrani0369@stu.kau.edu.sa.

^2^ Center of Research Excellence in Artificial Intelligence and Data Science, King Abdulaziz University, Jeddah 21589, Saudi Arabia

^3^ Center of Excellence in Genomic Medicine Research (CEGMR), King Abdulaziz University, Jeddah 21589, Saudi Arabia

^4^ Centre of Artificial Intelligence in Precision Medicines, King Abdulaziz University, Jeddah 21589, Saudi Arabia

^5^ Computer and Electrical Engineering Department, King Abdulaziz University, Jeddah 21589, Saudi Arabia; [rghneim@kau.edu.sa](mailto:rghneim@kau.edu.sa)

^6^ John Hopkins Aramco Healthcare, Dhahran, Saudi Arabia; ali.gac@gmail.com

## S1. Medical Guidelines


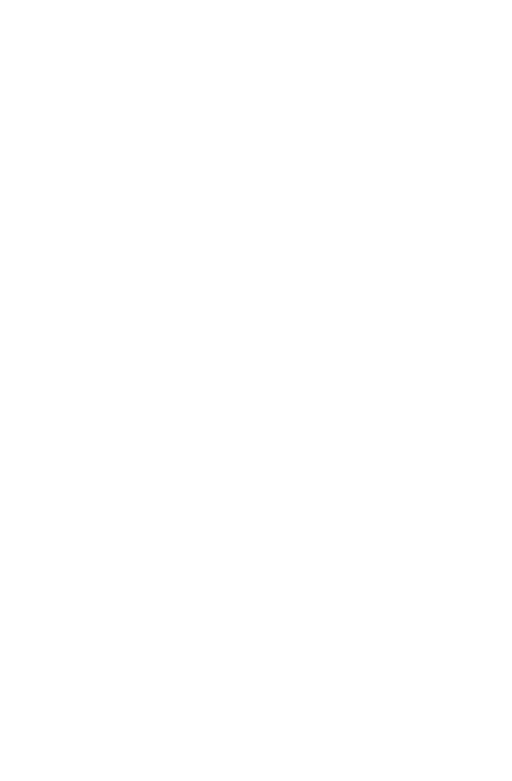

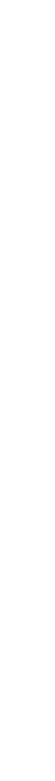

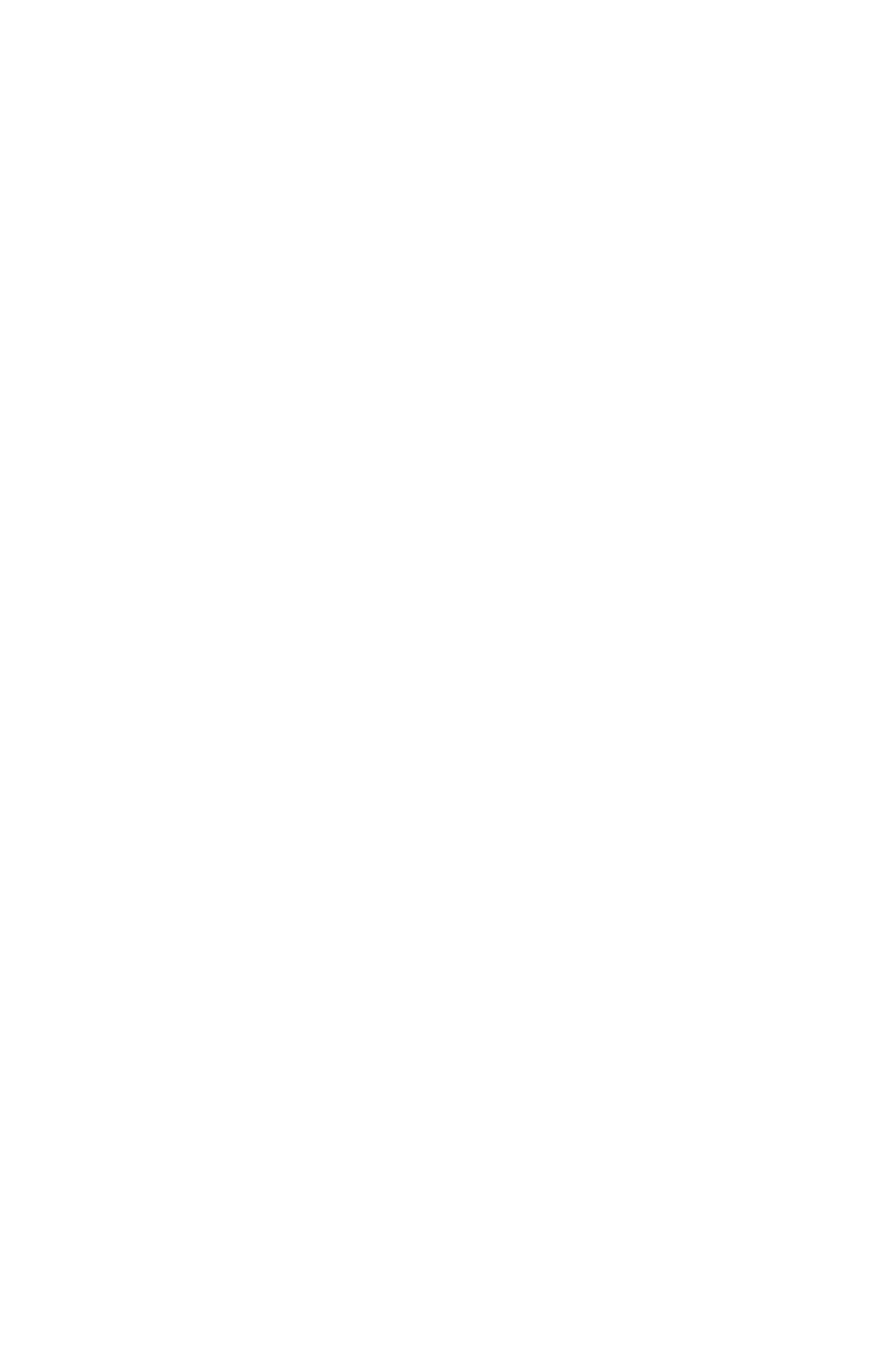

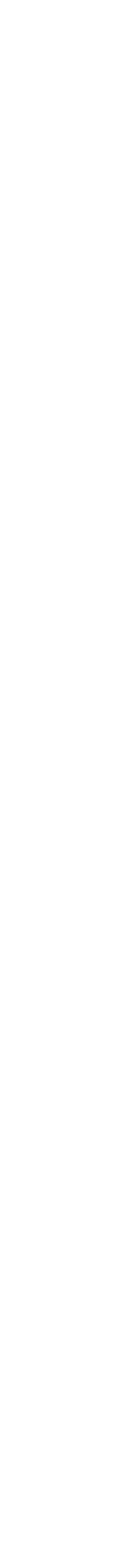


### 1.1 Injury, Symptoms, Risk Factors, Mechanisms, and Recommendations

| **Injury** | **Symptom, Risk Factors, and Mechanisms** | **Recommendation** |
| --- | --- | --- |
| **Ankle Fracture** | - Joint swelling  - Pain  - Deformity of ankle  - Laceration  - Ecchymosis  - Discoloration of the skin  - Smoking  - Elevated BMI  - Osteoporosis  - Absent pulse  - Tenderness to palpation of the lateral malleolus or the posterior, distal 6cm of the fibula  - Tenderness to palpation of the medial malleolus or the posterior, distal 6cm of the tibia  - Inability to bear weight for four steps | - Avoid weight  -bearing on the injured foot  - Cover any wound with sterile gauze or cloth  - Elevate the leg and apply ice to the swollen area |
| **Description** | An ankle fracture affects the medial or posterior malleolus of the tibia and/or the lateral malleolus of the fibula.  There are various types of ankle fractures (Weber A, Weber B, Weber C), and the severity varies depending on the specific characteristics of each fracture. |  |

| **Injury** | **Symptom, Risk Factors, and Mechanisms** | **Recommendation** |
| --- | --- | --- |
| **Ankle Sprain** | - Ankle twist  - Immediate swelling and pain (minor, moderate, severe)  - Bruising  - Skin discoloration  - Difficulty walking  - Weight-bearing issues (minimal to severe pain) | - Keep the ankle elevated above heart level to reduce swelling  - Apply ice to the swollen area; avoid prolonged icing |
| **Description** | An ankle sprain involves an inversion-type twist causing ligament damage. It is a common injury treated in emergency departments. |  |

This table summarizes the symptoms, risk factors, and recommendations for managing ankle fractures and sprains. It highlights the importance of proper diagnosis and treatment to ensure effective recovery.


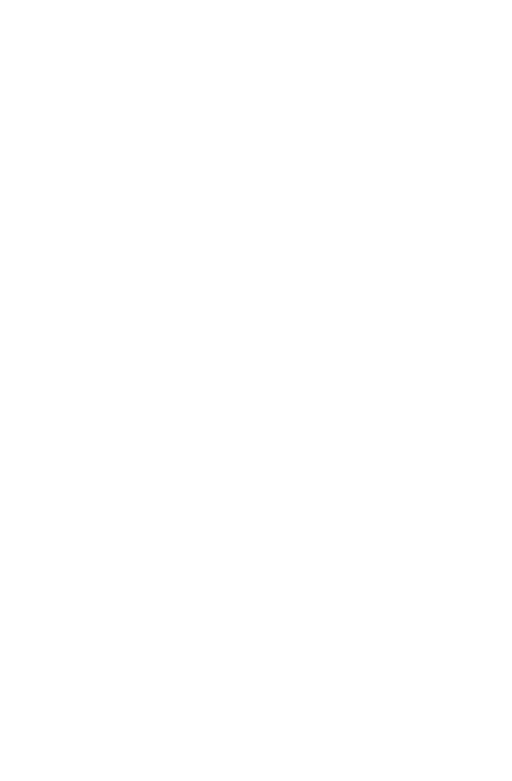

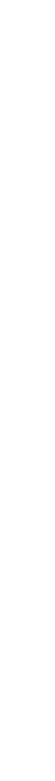

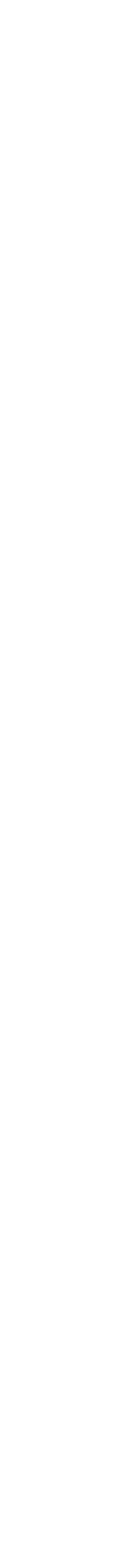


### 1.2 Recovery Plan for Weber A

| **Weeks** | **Recovery Plan** |
| --- | --- |
| **0-4 weeks** | - Wear the boot with TENS at all times when walking; no need to wear it in bed or when resting at home.  - Use crutches to offload weight as required.  - Start Stage 1 Exercises & Advice. |
| **4-6 weeks** | - Gradually discontinue the use of the boot and crutches; begin walking around the house without them.  - Wear the boot with TENS for longer distances outdoors.  - Move on to Stage 2 Exercises. |
| **6-12 weeks** | - Fracture should be largely healed.  - Gradually resume normal activities as pain allows; heavier tasks may still cause discomfort and swelling.  - Begin Stage 3 Exercises if needed. |
| **12+ weeks** | - Continued improvement in symptoms expected over the next few months.  - If significant pain or stiffness persists, consult healthcare provider for further advice. |

### 1.3 Recovery Plan for Weber B

| **Weeks** | **Recovery Plan** |
| --- | --- |
| **0-6 weeks** | - Wear the boot with TENS at all times when walking; it is not necessary to wear it in bed or when resting at home.  - Use crutches to offload weight as needed.  - Start Stage 1 Exercises & Advice. |
| **6-8 weeks** | - Gradually stop using the boot and crutches; start by walking around the house without them.  - Wear the boot with TENS for longer distances outdoors.  - Move on to Stage 2 Exercises. |
| **8-12 weeks** | - Fracture should be largely healed.  - Gradually resume normal activities as pain allows; heavier tasks may still cause discomfort and swelling.  - Continue with Stage 3 Exercises if needed. |
| **12+ weeks** | - Continued improvement in symptoms expected over the next few months.  - If significant pain or stiffness persists, consult healthcare provider for further advice. |

These tables outline the recommended recovery plans for Weber A and Weber B fractures, detailing the progression through different stages of healing and rehabilitation. The focus is on gradually increasing activity levels while managing symptoms and ensuring proper healing.

## 1.4 Stage 1 Exercises

| Exercises | Explanation |
| --- | --- |
| \| Point your injured foot up and down within a comfortable range of movement. Repeat 10 times. \| \| --- \| | 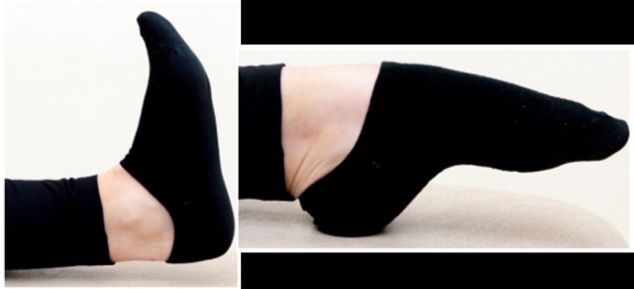 |
| Turn your foot outwards, leading with the outer edge (little toe), and then inwards, leading with the inner edge (big toe). Keep the rest of your leg still. Repeat 10 times. | 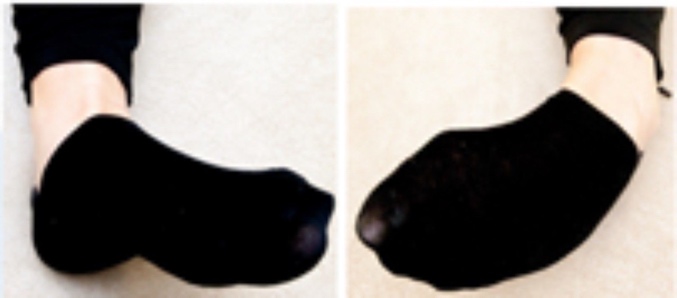 |
| Make circles with your foot in one direction, then switch and repeat in the other direction. Repeat 10 times. |  |

## 1.5 Stage 2 Exercises (3-4 times a day)

**1.5.1 Balance Exercises**

| Exercises | Explanation |
| --- | --- |
| 1. Sit with your injured leg  straight out in front of you. Place a towel or dressing gown cord around your foot  and pull it towards you until you feel a stretch in the back of your calf. Hold for 30 seconds. Repeat 3 times. | 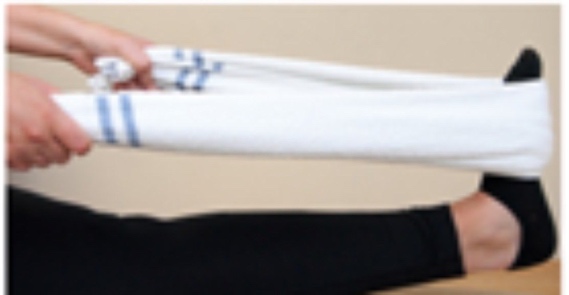 |
| 2. Sit on a chair. Cross your  injured ankle over your other knee. Place your hand over the top of your ankle and stretch the top of your foot and ankle, pointing your toes downwards. Hold for 30 seconds. Repeat 3 times.  OR Place your foot on a chair behind you, as shown in the picture. Hold onto something for balance. Point your foot and ankle downwards. You should feel a stretch at the front of your foo& ankle.  Hold for 30 seconds.Repeat 3 times. | 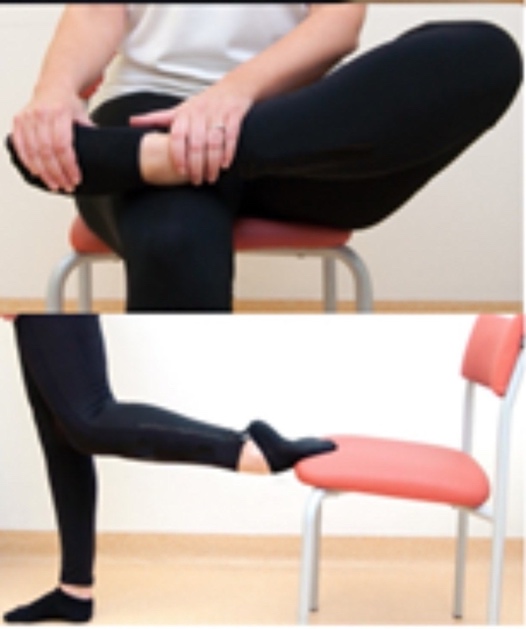 |

**1.5.2 Stretches Exercises**


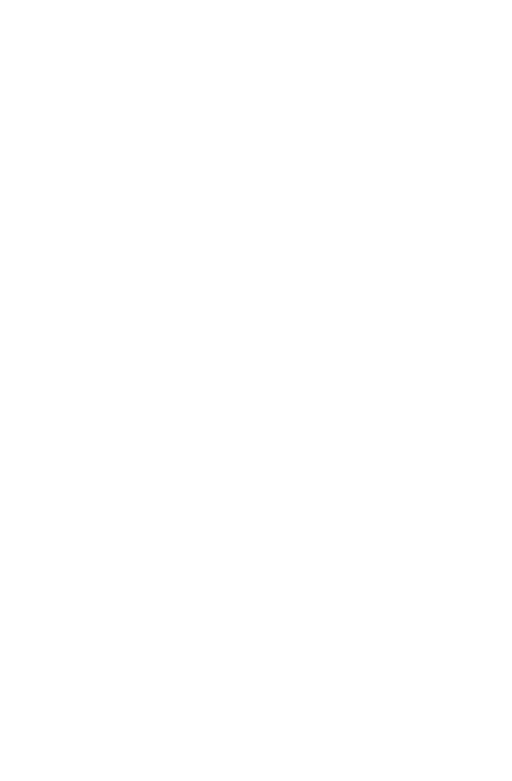

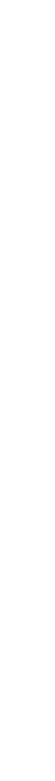

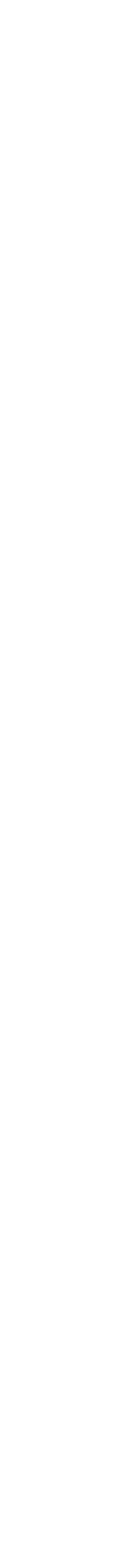


| Exercises | Explanation |
| --- | --- |
| Balance Exercise Level 1  1. **Level 1a:** Stand with your feet together and hold onto something for balance. Maintain this position for 30 seconds. If you can do this comfortably, move on to Level 1b. 2. **Level 1b:** Stand with your feet together without holding onto anything. Balance in this position for 30 seconds. If you can do this without difficulty, proceed to Level 1c. 3. **Level 1c:** Place one foot in front of the other, as close as you comfortably can. Hold onto something for balance if needed and maintain the position for 30 seconds. Repeat with the other foot in front. If you can do this comfortably and safely, try balancing without holding onto anything. | 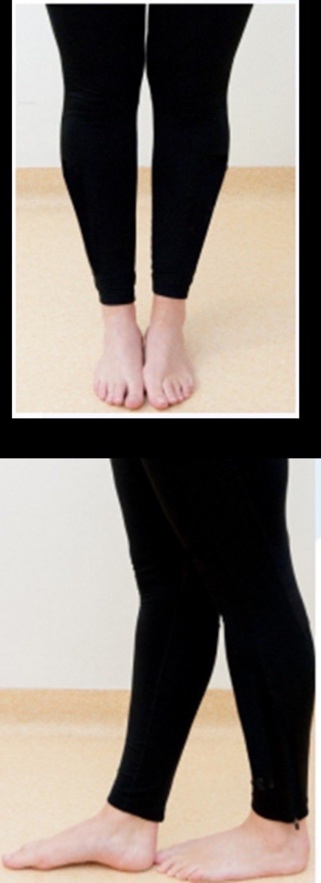 |
| Balance Exercise Level 2  1. **Level 2a:** Hold onto a firm surface for balance and aim to stand on your injured leg, ensuring it doesn't increase your pain. Maintain this position for 30 seconds. Once you can do this pain-free, progress to Level 2b. 2. **Level 2b:** Stand on your injured leg without holding onto anything for balance. Hold this position for 30 seconds. If you can achieve this, move on to Level 2c. 3. **Level 2c:** Attempt to perform the exercise with your eyes closed. Always stand in a safe environment with a firm surface nearby in case you need it. Hold this position for 10 seconds. | 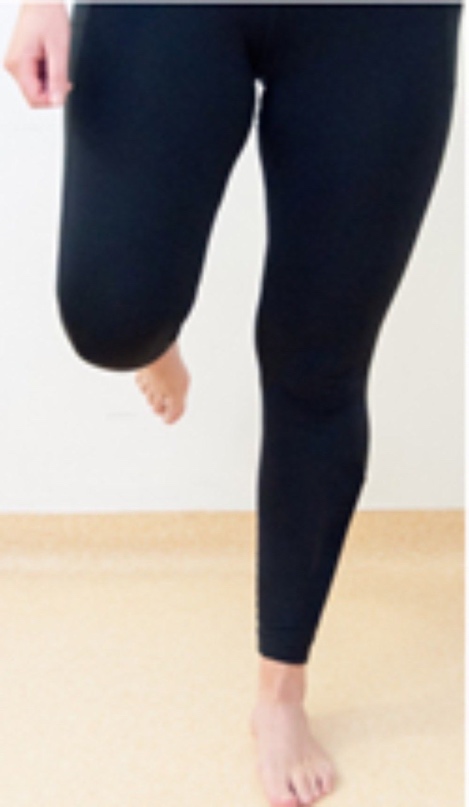 |

**1.5.3 Heel Raises**

As shown in figure 1, stand and then hold on to a stable object for support. Push up onto your toes, lifting your heels. Repeat 10 times.


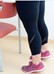


Figure 1. Heel Raises exercise.

## 1.6 Stage 3 Exercises (3-4 times a day)

These are optional advanced exercises designed for sports rehabilitation.

| Exercises | Explanation |
| --- | --- |
| Balance Exercise Level 1  1. **Level 1a:** Stand on your injured leg on an uneven surface, such as a pillow or a wobble board/cushion. Aim to maintain this position for 30 seconds. Once you can do this without pain, proceed to exercise Level 1b. 2. **Level 1b:** Attempt the same exercise with your eyes closed. Ensure you are in a safe environment with a firm surface nearby for support if needed. Try to balance for 10 seconds. | 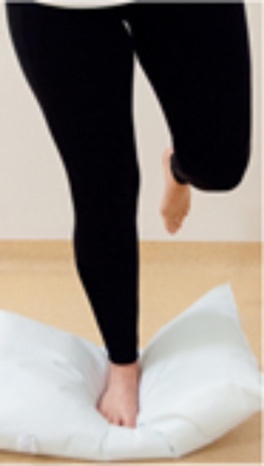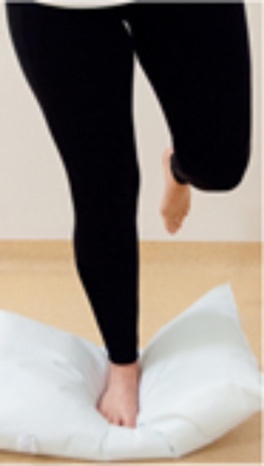 |
| Balance Exercise Level 2  1. **Level 2a:** Stand on your injured leg with your hands together. Swing your arms in a figure-eight motion in both directions for 30 seconds. 2. **Level 2b:** Perform the same exercise with your eyes closed. Aim to maintain your balance for 10 seconds. | 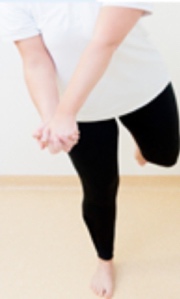 |

## S2. Relational Database schema


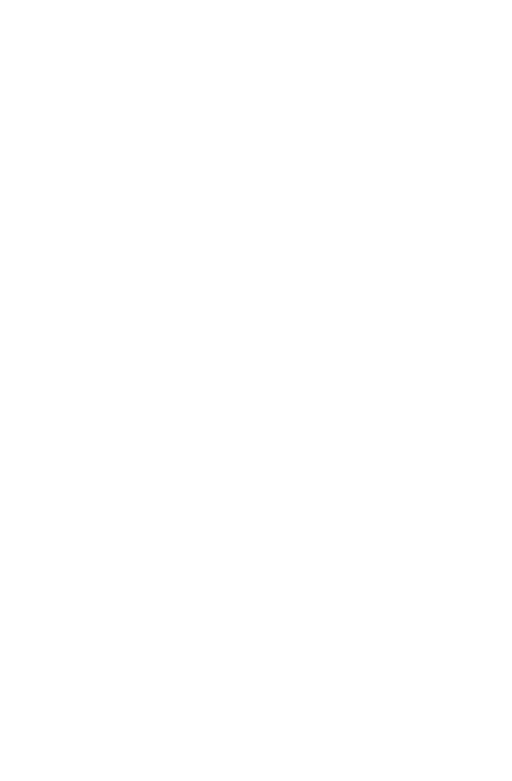

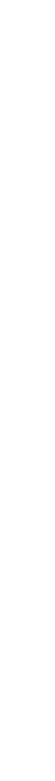

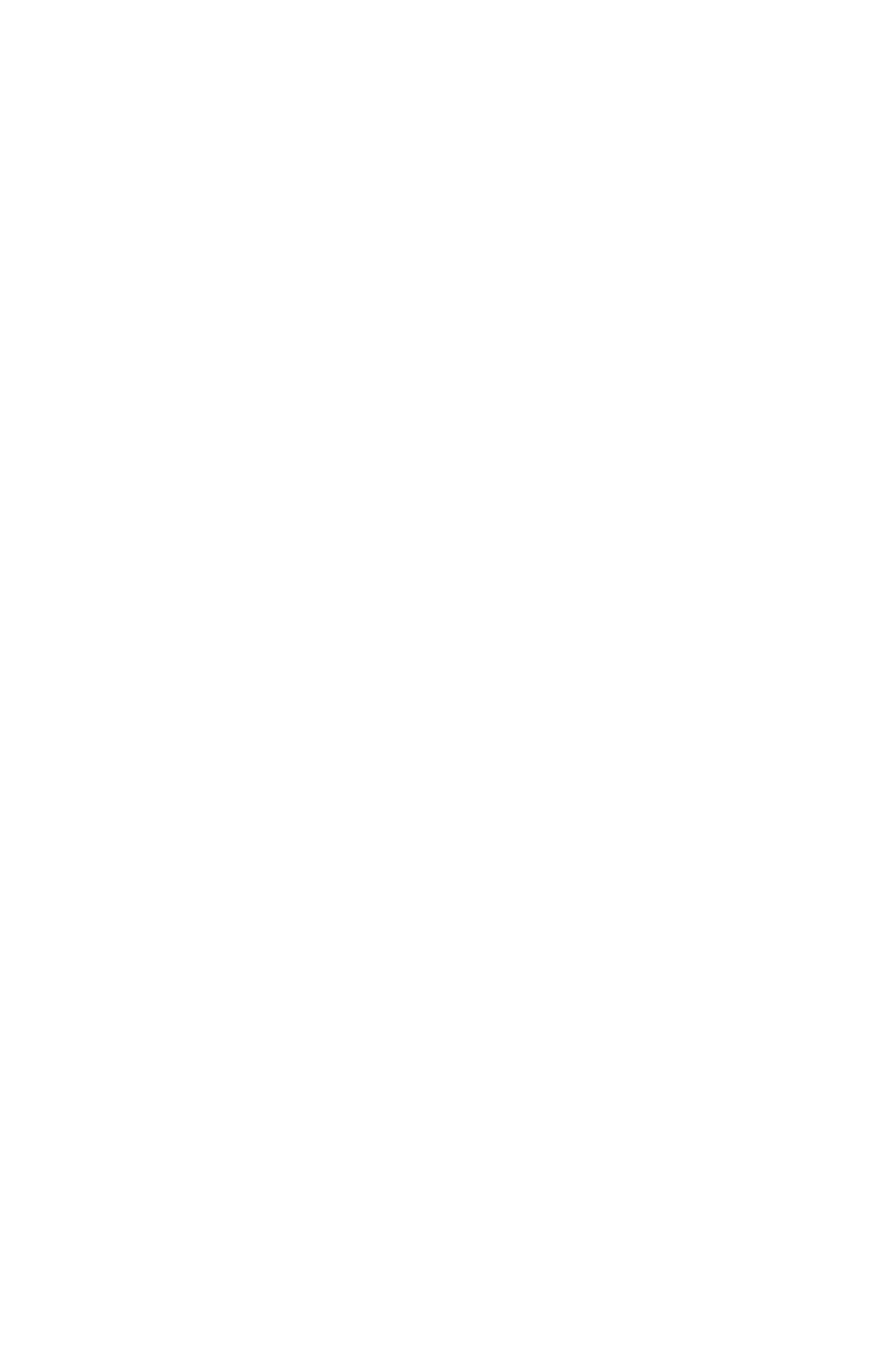

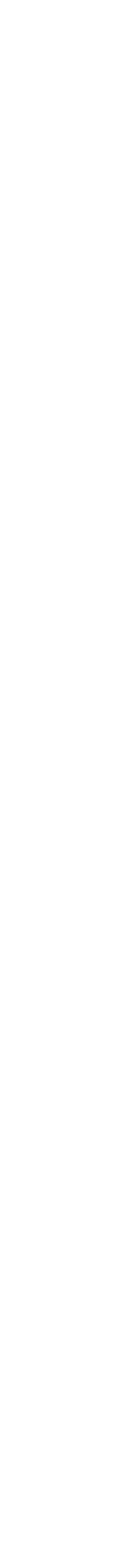


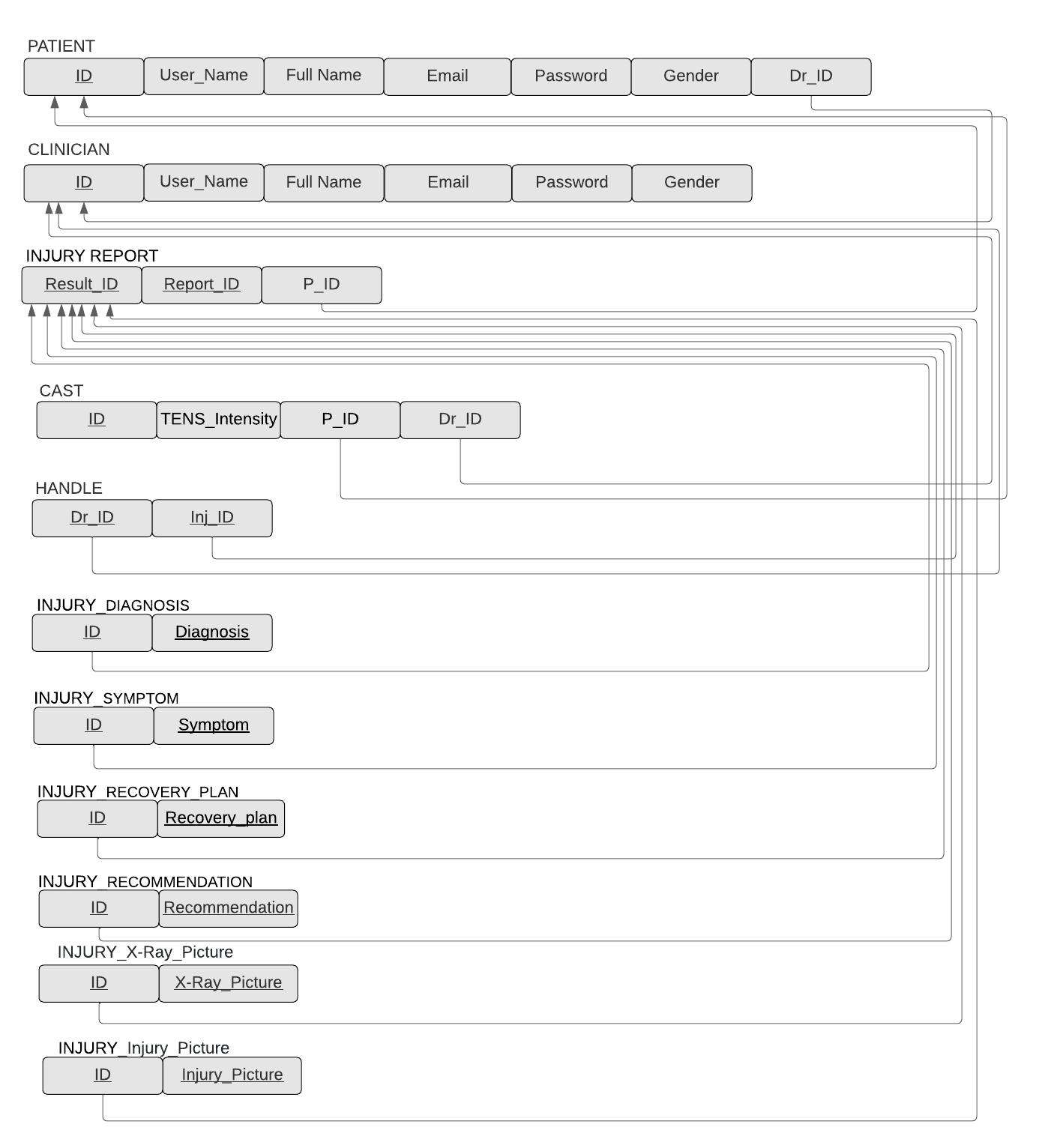


Figure 2. Database Diagram

## S3. Flowchart Diagram of Patient’s Authorities


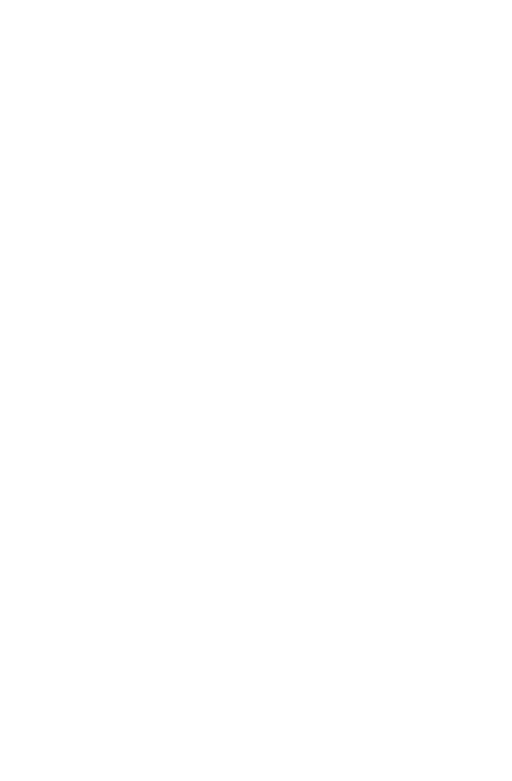

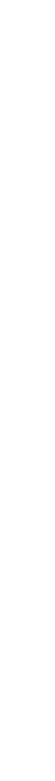

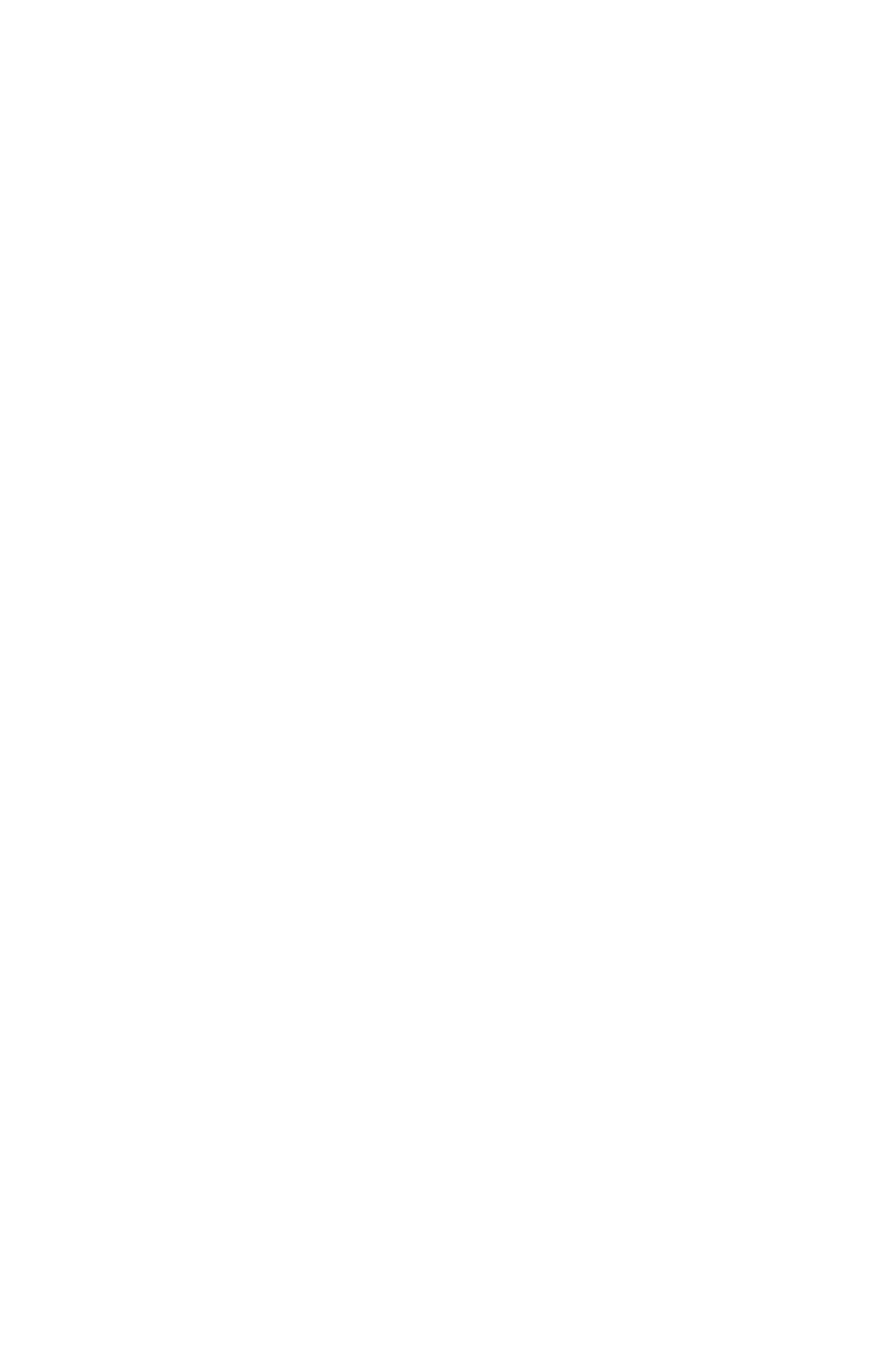

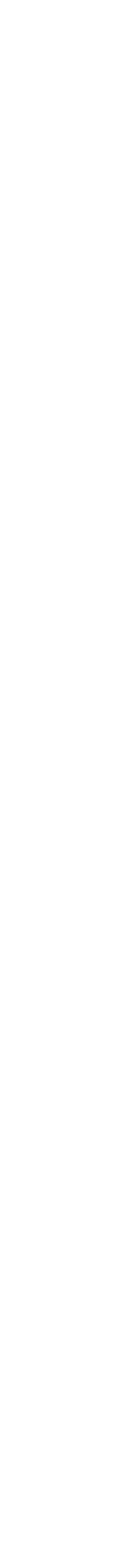


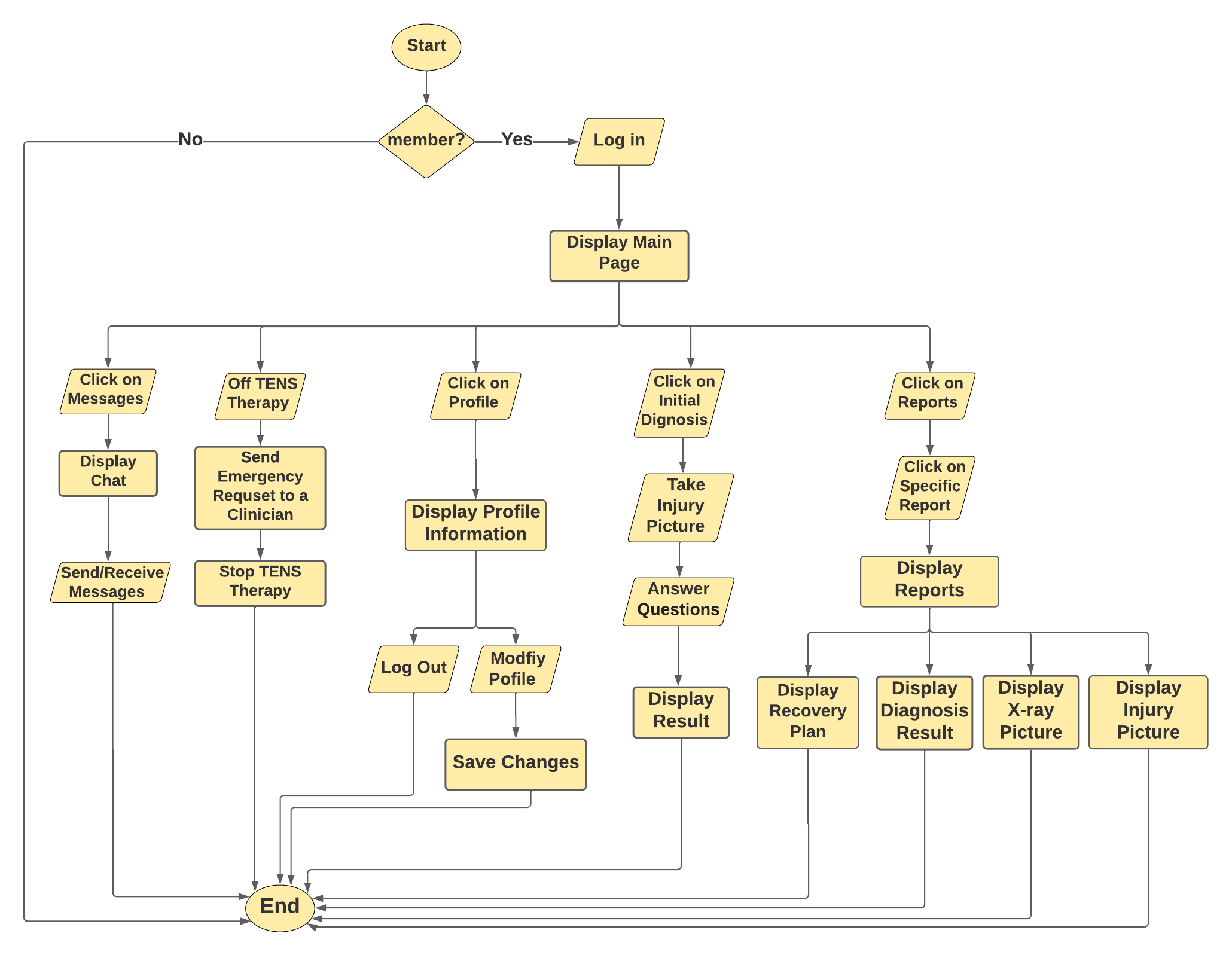


Figure 3. Flowchart Diagram

## S4. Data Flow Diagram (DFD)


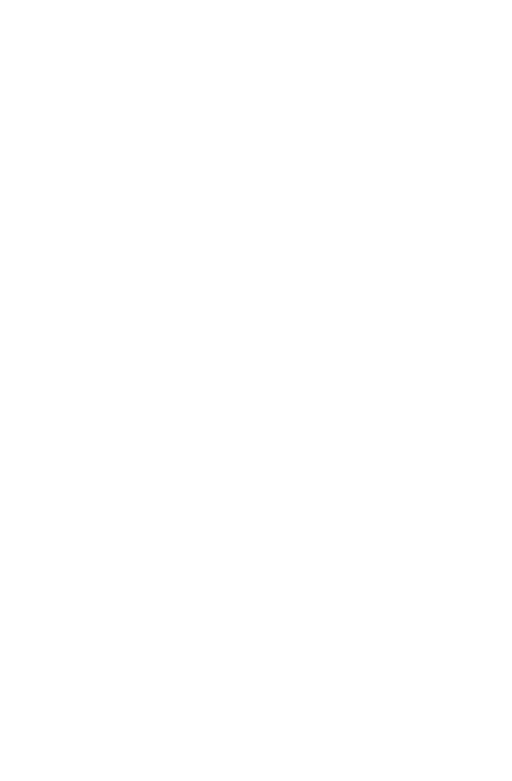

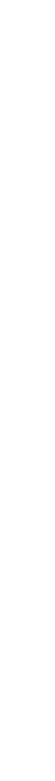

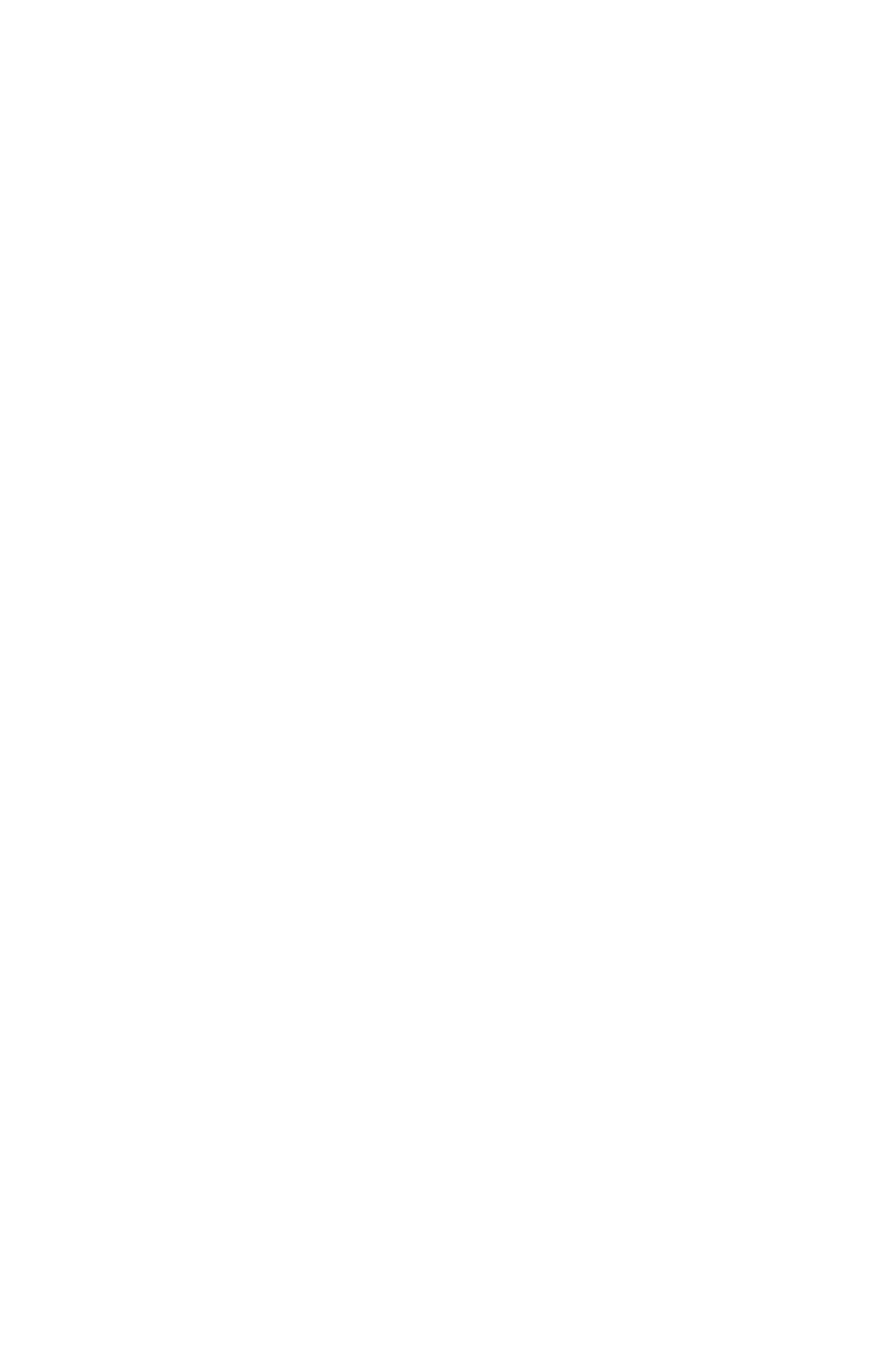

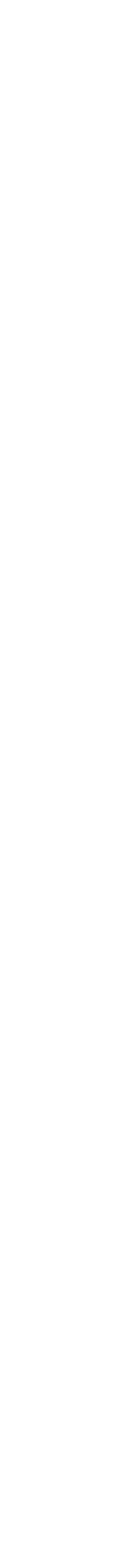


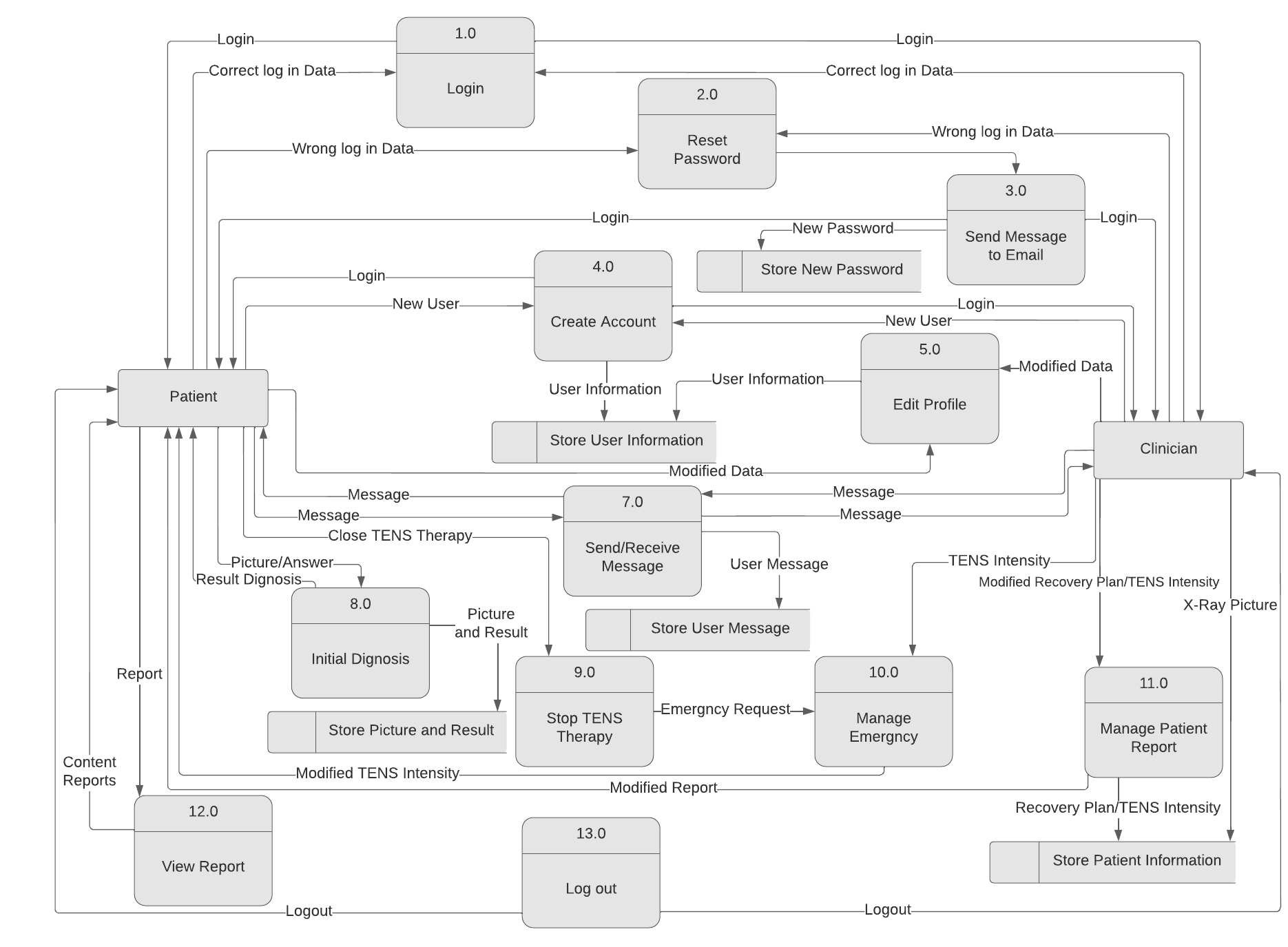


Figure 4. DFD Diagram

**S5. Usability and User Satisfaction**

| Task | Completion Time (sec) | Error Rate | User Feedback |
| --- | --- | --- | --- |
| Create Account (User A) | 36 | 0% | Excellent |
| Create Account (User B) | 27 | 0% | Excellent |
| Login | 39 | 0% | Excellent |
| Initial Diagnosis (User A) | 638 | 0% | Acceptable |
| Initial Diagnosis (User B) | 531 | 0% | Acceptable |
| Edit Profile (User A) | 35 | 0% | Excellent |
| Edit Profile (User B) | 32 | 0% | Excellent |
| Send Message (User A) | 52 | 0% | Excellent |
| Send Message (User B) | 56 | 0% | Excellent |
| Reset Password (User A) | 31 | 0% | Excellent |
| Reset Password (User B) | 29 | 0% | Excellent |
| View Reports | 33 | 0% | Excellent |
| Upload Injury Picture (User A) | 41 | 0% | Excellent |
| Upload Injury Picture (User B) | 37 | 0% | Excellent |
| Logout (User A) | 38 | 0% | Excellent |
| Logout (User B) | 39 | 0% | Excellent |


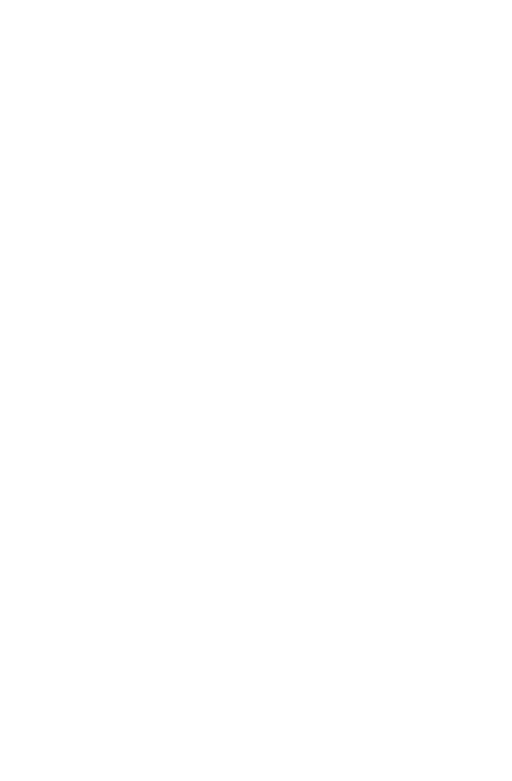

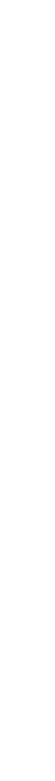

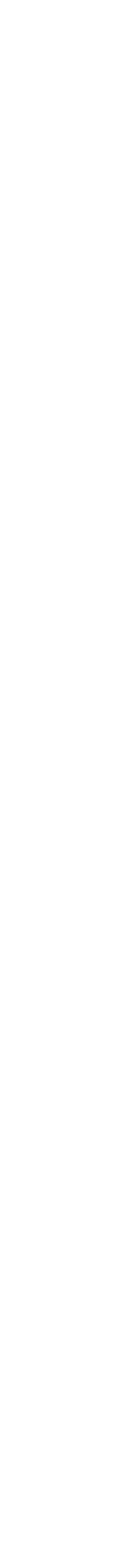

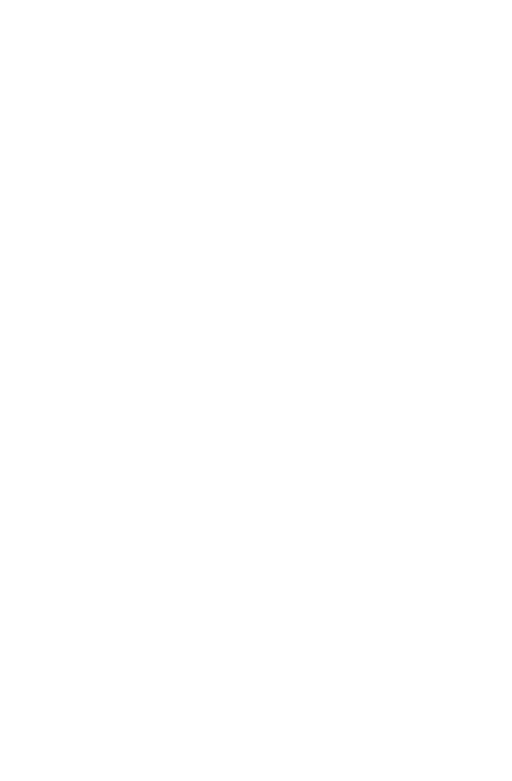

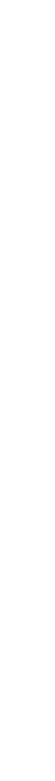

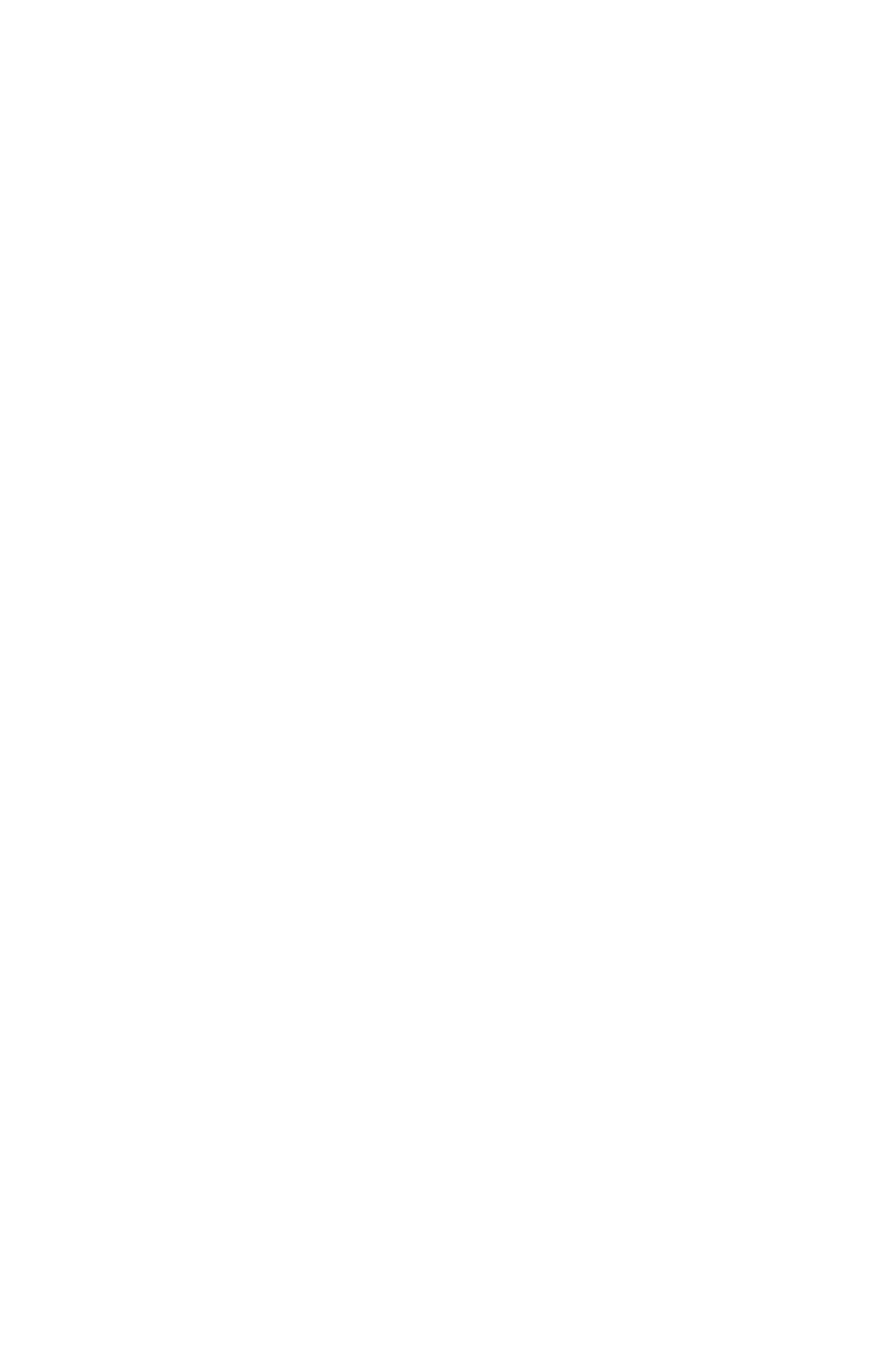

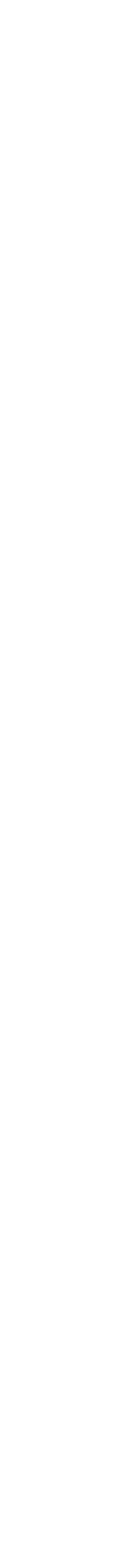

Supplement: Supporting Information — Additional supporting information can be found online in the Supporting Information section. S1: Medical guidelines. S2: Relational database schema. S3: Flowchart diagram of patient's authorities. S4: Data flow diagram. S5: Usability and user satisfaction. [file 5878245.f1.docx]
